# Robust, Long‐Term, and Exceptionally Sensitive Microneedle‐Based Bioimpedance Sensor for Precision Farming

**DOI:** 10.1002/advs.202101261

**Published:** 2021-06-17

**Authors:** Abdullah Bukhamsin, Khalil Moussi, Ran Tao, Gilles Lubineau, Ikram Blilou, Khaled Nabil Salama, Jürgen Kosel

**Affiliations:** ^1^ Biological and Environmental Science and Engineering Division (BESE) King Abdullah University of Science and Technology (KAUST) Thuwal 23955 Saudi Arabia; ^2^ Computer, Electrical and Mathematical Sciences and Engineering Division (CEMSE) King Abdullah University of Science and Technology (KAUST) Thuwal 23955 Saudi Arabia; ^3^ Physical Science and Engineering Division (PSE) King Abdullah University of Science and Technology (KAUST) Thuwal 23955 Saudi Arabia; ^4^ Sensor Systems Division (SeS) Silicon Austria Labs (SAL) Villach 9524 Austria

**Keywords:** impedance biosensor, micromolding, microneedles, precision farming, smart farm

## Abstract

Precision farming has the potential to increase global food production capacity whilst minimizing traditional inputs. However, the adoption and impact of precision farming are contingent on the availability of sensors that can discern the state of crops, while not interfering with their growth. Electrical impedance spectroscopy offers an avenue for nondestructive monitoring of crops. To that end, it is reported on the deployment of impedimetric sensors utilizing microneedles (MNs) that can be used to pierce the waxy exterior of plants to obtain sensitive impedance spectra in open‐air settings with an average relative noise value of 3.83%. The sensors are fabricated using a novel micromolding and release method that is compatible with UV photocurable and thermosetting polymers. Assessments of the quality of the MNs under scanning electron microscopy show that the replication process is high in fidelity to the original design of the master mold and that it can be used for upward of 20 replication cycles. The sensor's performance is validated against conventional planar sensors for obtaining the impedance values of *Arabidopsis thaliana*. As a change is detected in impedance due to lighting and hydration, this raises the possibility for their widespread use in precision farming.

## Graphical abstract

A microneedle electrode is developed, based on a scalable, versatile, and high‐resolution fabrication process for minimally invasive impedimetric plant biosensors. The metal‐coated polymeric microneedles can pierce the waxy coating layer of the plant tissue to establish direct contact with the extracellular fluid and assess its physiological state via impedance. A demonstration of the sensor's performance is conducted on *Arabidopsis thaliana*.

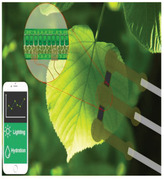

## 1 Introduction

To keep up with the constantly growing global demand for food, farmers are forced into adopting technologies to uniformly manage large fields. While this approach yields many economy‐of‐scale benefits, as the quantity of arable land is exhausted, due to resource constraints, production growth may plateau, potentially leading to a surging nutritional deficit. To keep pace with the rising demands of a growing population, the food production capacity must double by 2050. Yet, studies have shown that the current yield rates are projected to fall short of this goal.^[^ ^1^ ^]^ This challenge is compounded by the significant environmental footprint of agriculture driving losses in biodiversity and climate change. It is estimated that about half of the habitable land and 70% of annual freshwater withdrawals are dedicated to agriculture alone.^[^ ^2^ ^]^ Against the backdrop of an impending food shortage, the development of precision farming (PF) techniques, which seek to link the real‐time needs of crops to the administered supply, has accelerated. By utilizing an array of technologies that span drones equipped with spectral cameras to soil‐implanted sensors, PF may enable farmers to address heterogeneities in their fields, thereby increasing their productivity with little to no increase in the resource footprint. The impact and adoption of PF can be greatly accelerated by the procurement of sensitive and accurate information regarding the physiological status of the crop.^[^ ^3^ ^]^ With that said, current commercially available options do not achieve this goal as it requires direct nondestructive monitoring of a plant's extracellular fluid (EF).^[^ ^3^ ^]^ Due to the high spatial and temporal heterogeneity of farm fields and their scale, soil‐implanted sensors are not suitable to accurately monitor crops either, as they tend to aggregate averaged information about the content of the soil, which may be correlated to but is not directly reflective of the EF status of the crop.^[^ ^4^, ^5^ ^]^ To circumvent this challenge, utilizing electrical impedance spectroscopy (EIS) on plant tissue has been a long‐standing subject of interest. Structural changes that occur in the plant, which reflect its physiological state, manifest in the tissue's ionic content, membrane permeability, or viscosity. As such, in effect, the use of EIS can allow for quick and nondestructive assessment of such structural changes and, by extension, the physiological state of the plant tissue.^[^ ^6^ ^]^ However, the utility of such an approach is limited by the lipid‐rich barrier layer, known as the cuticle that encapsulates the above‐ground exterior of plants. Such layers act as large electrical insulators that effectively mask the underlying physiologically relevant impedance. This is especially troublesome at the lower frequency range where electrode polarization (EP) is at its highest. Polarization occurs due to the buildup of an electric double layer of ions at the interface between the electrode and the electrolytes in the tissue. It results in a potential drop of the electrode relative to its value in an open‐circuit configuration by behaving as an additive impedance. As the EP has been noted to decrease with frequency via power‐law relation by capacitive shorting, at low frequencies below 1 kHz, it can contribute significantly to the measured impedance.^[^ ^7^ ^]^ Borrowing a page from medicine,^[^ ^8^ ^]^ minimally invasive microneedles (MNs) were developed to directly probe the relevant plant tissue, thereby bypassing the cuticle. As this approach gained traction, MNs were used for DNA extraction from leaves,^[^ ^9^ ^]^ EIS measurement of plant tissue to probe the content of the EF^[^ ^10^, ^11^ ^]^ and to assess sap flow through the plant's stem.^[^ ^12^ ^]^

Due to the breadth of their applications, several MN fabrication techniques were devised. Earlier iterations relied on existing micromachining strategies of Silicon^[^ ^13^ ^]^ or serial approaches such as backside exposure.^[^ ^14^ ^]^ Even the first to market MNs‐based products were fabricated using serial processes such as laser ablation of metal. Conventionally, anisotropic wet etching and isotropic dry etching methods have been combined to yield more control over the MN geometry, but these processes are often labor‐intensive and are limited in their applicability to a limited set of materials. Alternative approaches had to be devised as the aforementioned techniques do not lend themselves to scalable production or often limit the achievable MN design. In pursuit of cost effective and batch manufacturing techniques for MNs, silicone micromolding using an MN array template has been extensively explored in literature. Silicone varnish can be used to fabricate elastomeric molds via hot‐embossing, which can then be subsequently used for scalable replication whilst not sacrificing the high fidelity of the master template. The elastomeric properties of these intermediary molds are crucial for the final release of the MNs. During demolding, a lateral bending force is applied to the mold to release the MNs. Using elastomeric molds ensures that the mold can be easily bent at a low bending force to avoid plastic deformation of the MNs. The versatility of the strategy allows it to be compatible with several thermosetting polymers, such as cycloolefin polymers^[^ ^15^ ^]^ and polyimide,^[^ ^16^ ^]^ organically modified ceramics,^[^ ^17^ ^]^ and UV‐curable polymers, such as SU‐8.^[^ ^18^ ^]^

As the replication fidelity of micromolding is high, the achievable geometry is limited only by the fabrication process used to generate the master template and the release technique for demolding. To fabricate the master template, both traditional micromachining strategies and additive manufacturing techniques, such as 3D printing, have been explored. Whilst the latter offers a high degree of control over the geometry of the MNs with high‐aspect ratios, it is subject to limits set by the resolution of the printer resolution. Although a two‐step “Print and Fill” method was devised to overcome this limit on low‐cost 3D printers,^[^ ^19^ ^]^ the MNs fabricated using such a technique have a tip radius of 20–40 µm, which increases the force needed for insertion. With the advent of two‐photon polymerization (TPP) based laser lithography, the gap between scalable production and control over MNs design and geometry can be bridged. With a resolution limit of 900 nm, MN master templates can be fabricated for micromolding.^[^ ^15^, ^20^ ^]^ However, regardless of the strategy employed for the fabrication of the master template, the lateral bending force applied for demolding considerably limits the achievable geometry. This is especially evident in the thickness of the support bed of the MNs used previously. For example, micromolded SU‐8 MNs utilize a 1 mm thick bed to ensure minimal deformation during demolding.^[^ ^18^ ^]^ The thickness of the support bed influences both the conformability and the weight of the array on soft tissue. The former is needed to ensure adequate contact and adhesion to the surface and the latter should be minimized to facilitate the application on delicate leaf structures.

**Figure 1:**
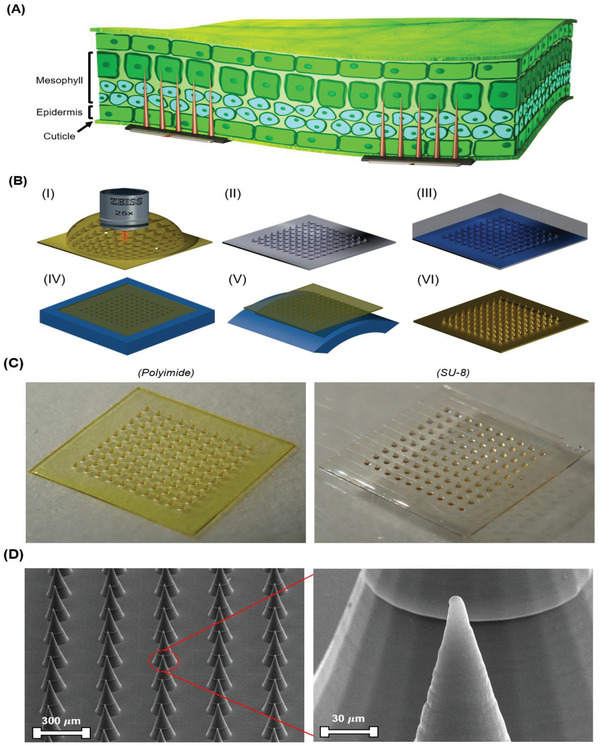
A) An illustration showing the impedimetric sensor attached to a plant leaf. B) Schematic illustration of the fabrication process of the MNs‐equipped electrodes: I) A master mold is fabricated using TPP laser lithography on a Silicon die. II) After development, the mold is coated with a 5 µm layer of Parylene C via chemical vapor deposition (CVD). III) Uncured PDMS (1:10, curing agent to monomer) is cast on top of the master mold and is cured at 90 °C for 45 min. IV) The SU‐8/polyimide varnish is cast onto the PDMS imprinted mold and is outgassed and appropriately cured. V) The mold is submerged in chloroform and is sonicated at a frequency of 45 kHz for 1 min. VI) The MNs are demolded and layers of 10 nm of titanium followed by 150 nm of gold are sputtered on the MNs. C) Image of polyimide (left) and SU‐8 (right) MNs after demolding. D) SEM images of polyimide MNs at two magnification levels.

To that end, we present an alternative micromolding release strategy for the fabrication of polymeric MNs combining the advantages of scalable production and a high degree of control over the achievable geometry, whilst not compromising on the mechanical properties of the MNs. In this proposed strategy, we employ an elastomeric intermediary mold, which is fabricated via the embossing of polydimethylsiloxane (PDMS) on a master mold fabricated using TPP‐based laser lithography. A photosensitive resist (SU‐8) or a thermosetting polymer (polyimide) is then cast over the intermediary mold to fill its conical cavities and subsequently baked or UV‐crosslinked. To gently release the polymeric MNs, the elastomeric molds are submerged in a chlorinated hydrocarbon solvent. This causes swelling in the PDMS matrix, which translates to volume expansion of the matrix. Upon expansion, the mold gently and gradually releases the MNs from the conical cavities, pushing them out. Combined with a short burst of gentle sonication, the MNs can be released with minimal bending force. We demonstrate how this strategy can be used to fabricate MNs. These MNs‐equipped electrodes are employed for EIS measurements of *Arabidopsis thaliana* for validation as a plant impedimetric sensor (**Figure** **1**a).

## 2 Results and Discussion

### 2.1 Fabrication and Characterization

The schematic outline of the fabrication process used to micromold the MNs is shown in Figure 1b. The use of PDMS (Sylgard 184) for the intermediary mold confers many advantages to the fabrication pipeline. As PDMS is largely transparent in the UV‐B (290–320 nm) and the UV‐A (320–400 nm) regions, it can be used with a host of photosensitive materials that crosslink in this bandwidth.^[^ ^21^ ^]^ Furthermore, the thermal stability of the PDMS matrix crosslinking allows for its use in molding thermosetting polymers up to a temperature of 250 °C, which can be elevated by the introduction of copolymers.^[^ ^22^ ^]^ Lastly, the stability of its mechanical properties for several months^[^ ^23^ ^]^ allows for the repeatable use of the elastomeric molds. Submersion of PDMS in chlorinated hydrocarbons, such as chloroform, swells the polymer matrix by up to 40%.^[^ ^24^ ^]^ The original shape of the mold can be restored postswelling by submerging it in a solution of diluted ethanol in deionized water (50% by volume). Upon submerging the PDMS mold in the solution, chloroform exits the polymer matrix. The miscibility of chloroform in ethanol allows it to remain in a unique phase that does not mix with water in solution. The PDMS molds can then be reused after being cleaned in isopropanol. This approach allows for scalable and high‐fidelity production of MNs that can be more easily integrated with a host of devices, which include but are not limited to electrochemical sensors, drug delivery platforms, and scaffolds for cell culture. Uniquely, it also allows for the fabrication of high aspect ratio free‐standing structures as thin as 65 µm, such as interdigitated arrays of MNs relevant for electrochemical sensing applications or shadow masks (Figure S1, Supporting Information). This negates the need to use complex metal‐pattern transferring techniques^[^ ^25^ ^]^ or laser ablation^[^ ^26^ ^]^ to pattern MN electrodes.

The master molds are fabricated using TPP‐based laser lithography using the photonic professional GT instrument (Nanoscribe, GmbH). IP‐S is chosen as the photoresist of choice due to its low shrinkage, mechanical properties, and adherence to Silicon. The master mold consists of an array of 100 MNs arranged with a spacing of 300 µm from tip to tip in a 10 by 10 configuration. The spacing is chosen to mitigate the bed‐of‐needles effect and to ensure that no MN will lie at the boundary of two or more rectangular slicing windows, which are set at a maximum size of 300 by 300 µm^2^ on the Describe software used to operate the instrument. Each MN in the array is conical in shape with a radius of 70 µm and a height of 350 µm. As the thickness of the MNs bed is 100 µm, one stitching line was included in the vertical span of the MNs. As the thickness of the cuticle varies between submicrometer to 10 µm depending on the species being probed and tissue hydration state,^[^ ^27^ ^]^ this length is more than adequate to span the cuticle even without full penetration of the MNs. A 5 µm thick layer of Parylene C is deposited on the master mold using a chemical vapor deposition process (Parylene LABCOATER, Specialty Coating Systems) to further anchor it to the Silicon substrate and to smooth the stitching lines between the slices for easier detachment of the PDMS after curing.

SU‐8 2005 (MicroChem) and polyimide PI‐2611 (DuPont) are chosen as photosensitive and thermosetting polymers for micromolding MNs. The crosslinking of SU‐8 can be initiated by exposure to 365 nm light whilst polyimide polymerizes at 200 °C, both of which are compatible with PDMS's optical transmission and thermal stability limit, respectively. The robust mechanical properties and biocompatibility of these polymers also allow for their use to penetrate the cuticle of living plants without triggering any cytotoxic effects.^[^ ^28^, ^29^ ^]^ More importantly, the low viscosity of the chosen formulations for SU‐8 and polyimide coupled with a relatively high solid content enables more flow into the conical cavities of the PDMS mold with little shrinkage after crosslinking or polymerization. As both polymers are dielectrics, they can be subsequently patterned with the use of shadow masks and direct metal sputtering.

The general processes for the fabrication of SU‐8 and polyimide MNs‐equipped electrodes are largely similar. The polymer varnish is cast onto the PDMS mold and is outgassed for 20–30 min to ensure that the polymer can flow into the cavities completely. The excess varnish is then removed using a clean surgical blade. For SU‐8, the MNs are then soft‐baked at 65 °C for 3 min. Subsequently, the temperature is raised to 95 °C and is held at 95 °C for another 5 min. After the sample has returned to room temperature, it is exposed to a 365 nm light source with an intensity of 14 mW cm^−2^ for 11 s. Finally, the SU‐8 MNs are baked again at 95 °C for 30 min to ensure complete crosslinking. For the polyimide MNs, after outgassing, the molds are baked on a hot plate at 90 °C for 1 min and 30 s and then baked at 150 °C for another 1 min and 30 s. After being allowed to cool to room temperature, the polyimide MNs are polymerized by baking them on a hot plate from room temperature to 200 °C where they are held for 45 min. Upon demolding, the SU‐8 and polyimide MNs can be annealed at 150 and 350 °C, respectively, for optimal mechanical performance. To assess the reliability of the master template during hot embossing of elastomeric molds, the template is used for several replication cycles in PDMS and was imaged by scanning electron microscopy (SEM). After the 32th replication cycle, the template demonstrably deforms, with the supporting layer curving upward from the Silicon die (Figure S2, Supporting Information). No visible cracks in the Parylene C film are observed and the template is believed to have failed due to repeated thermal loading. As a convection oven is used for embossing, the thermal gradients applied on the master template for curing and demolding PDMS are large. This may have caused thermal stress eventually leading to the deformation of the mold. This is further corroborated by the nearly intact state of the MNs in the master template after deformation. As the supporting layer relieves the built‐up thermal stress by arcing upward, the MNs in the array appear to remain largely intact.

The polymerized MNs are inspected under light microscopy to verify their integrity after demolding (Figure 1c). Both SU‐8 and polyimide MNs are also inspected under SEM to assess the fidelity of the replication process (Figure 1d and Figure S3, Supporting Information). The SEM images show that the master mold features are preserved upon replication, including the slicing lines resulting from TPP‐based laser lithography. As the stitching lines are 500 nm thick, this suggests that the replication process is high in its fidelity to the original design. This is reflected in the SEM images of both the SU‐8 and polyimide MNs. Although the morphology of SU‐8 and polyimide MNs is largely similar, the higher viscosity of the polyimide varnish manifests in more prominent stitching lines and a more curved MN tip. However, examining the tip geometry of the polyimide MNs under SEM with a higher level of magnification shows that the tip of the conical MNs is roughly spherical with an approximate diameter of 0.5 µm. This sharpness of the tip aids the penetration of the probed plant tissue. The prominent stitching lines do not impair the mechanical ability of the MNs to pierce a plant leaf. Likewise, the stitching gap does not result in a discontinuity in the sputtered metal layer as is confirmed by probe contact testing. Tilt‐corrected point measurements are used to characterize the MNs' shank height, base radius, and the thickness of the supporting bed (Figure S4, Supporting Information). In stark contrast to the matching average shank height and base radius of polyimide and SU‐8 MNs, the support layer thickness differs significantly. The SU‐8 MNs retain a 131 ± 5 µm thick supporting layer compared to 105 ± 4 µm for the polyimide MNs. This discrepancy is believed to be due to the higher solid content of the SU‐8 varnish relative to that of the polyimide varnish.

Crucially, the repeatability of the replication fidelity of the release process was assessed by inspecting the demolded MNs after several release cycles (Figure S5, Supporting Information). The same PDMS intermediary mold was used for 25 replications of the MNs. SEM inspection of the released MNs shows that the geometry of the released MNs begins to deviate from the intended design slightly at the 10th replication cycle, especially around the vertical stitching line. The stitching line represents a mechanical joint that is more susceptible to deformation as it connects the MNs tip to its base and, as such, is the first part to deform. Significant deformities that impair the piercing ability of the MNs by changing the shank length occur at the 25th replication cycle, rendering the elastomeric mold obsolete. The tips of the MNs are dislodged into the elastomeric mold at the stitching line, which behaves as a fault line during the mold swelling event before release. With that said, the consistency of the elastomeric mold over multiple replication cycles is in part due to the stable elastomeric properties of PDMS and in part due to the use of a gentle release mechanism, which applies a minimal lateral bending force on the MNs. In the same vein, to assess the success rate of the release process, 15 new PDMS molds are embossed from the same master template. Each mold is used to replicate and subsequently release polyimide MNs. This is done to ensure that any deformities observed are due to the release process and not due to the gradual degradation of the PDMS molds. The cured MNs are inspected using an SEM for any sign of deformation or breakage. While no breakage was observed, bent tips are observed in three of the released MNs (Figure S6, Supporting Information). This yields a success rate of 80% for the reported methodology.

**Figure 2:**
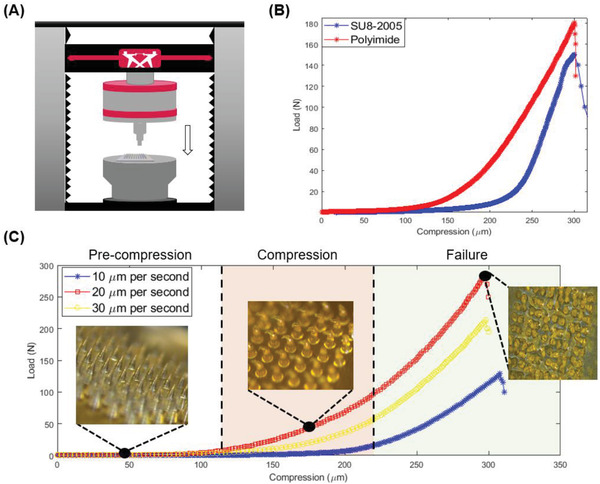
A) An illustration showing the mechanical testing setup used for the crash test. B) Load versus compression curve recorded for the compression of polyimide (red) and SU‐8 (blue) MNs during the crash test. C) Load versus compression curve recorded for polyimide MNs at compression rates of 10 (blue), 20 (red), and 30 µm s^−1^ (yellow). Insets show the image of the polyimide MNs at each stage.

To ensure that the MNs can pierce the cuticle, the MN arrays of SU‐8 and polyimide were tested in a crash‐test configuration shown in **Figure** **2**a. Using a 1 kN load cell, a flat metal bed was pushed against the MNs at a rate of 5 µm s^−1^ using the Instron 5966 mechanical testing instrument. The load and compression data were recorded for each sample until failure either by buckling or bending of the MNs. The load‐cell transducer was set to stop the test after a 40% drop from the peak load, which suggests that the failure of the MNs has occurred. Samples in which not all the MNs in the array failed were discarded. To verify failure, the MNs samples were imaged using a microscope at 16x magnification (LEICA M205 C) before and after compression. Normalizing the failure force to the MNs number in the array yields a force of 1.5 ± 0.13 and 1.8 ± 0.06 N per needle for SU‐8 and polyimide, respectively. The peak load was sustained for 5 and 11 µm of compression for polyimide and SU‐8 MNs, respectively. As the polyimide MNs proved to be more mechanically resilient, subsequent tests were not performed on the SU‐8 MNs. The crash test was repeated on the polyimide MNs with 10, 20, and 30 µm s^−1^ compression rates (Figure 2b). The failure load was determined to be sensitive to the rate of compression. Although the literature on the piercing force needed to pierce the cuticle is sparse, using the piercing force reported on hydrated Cacao leaves (*Theobroma cacao* L.) as a reference, which is 0.8 N,^[^ ^30^ ^]^ the polyimide MNs should be able to withstand the piercing event.

**Figure 3:**
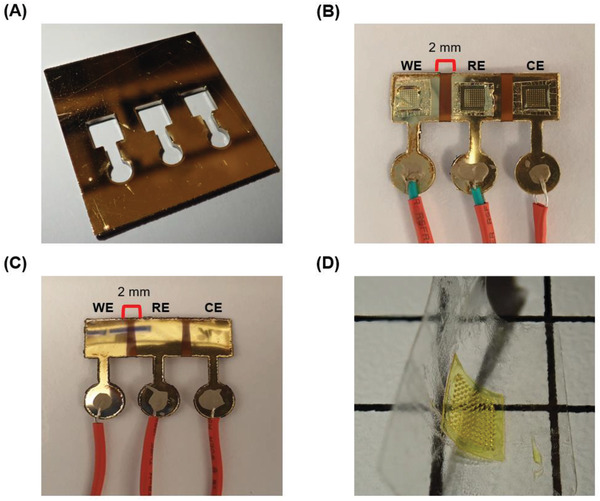
A) An image of the laser cut acrylic shadow mask used to pattern the sensor. B) A top view of the MNs‐equipped sensor showing the arrangement of the working electrode (WE), reference electrode (RE), and counter electrode (CE). C) A top view of the equivalent planar sensor with the same arrangement of electrodes. D) An image showcasing the flexibility of the polyimide MNs attached to a PDMS bed as they are deformed.

Prior to deployment on the plant, the polyimide MNs are placed on a single‐sided polished Silicon die (25 mm by 25 mm) covered by spin coating a 50 µm layer of uncured polyimide varnish. The die is heated to 350 °C to adhere the MNs to the film via cross polymerization. A CO_2_ laser is used to cut the film with the desired shape of the electrode bed on top of the Silicon die. The film is then subsequently peeled off the die to utilize its flexibility. To confer electrical conductivity, the MNs are coated with a layer of 10 nm of titanium followed by 150 nm of gold using sputter deposition. A laser‐cut poly(methyl methacrylate) shadow mask is used to ensure that each MN‐equipped electrode in the sensor was spaced by 2 mm to the closest neighboring electrode (**Figure** **3**a). To make the electrodes interconnects, silver conductive paste (Sigma‐Aldrich) is cast on exposed copper wires that are threaded through the film at the electrode junctions and cured at 150 °C for 30 min (Figure 3b). An equivalent planar electrode is fabricated using the same process, but without the addition of the MNs to assess the advantages of their integration (Figure 3c). As the bed and the molded MNs are flexible (Figure 3d), the sensor can fold onto the tissue being probed, including the stem and the leaf. The combined weight of the sensor with the attached wires is 186 mg rendering it suitable for use on a plant leaf with minimal deflection. Minimizing the weight of the sensor is crucial to mitigate triggering the thigmomorphogenetic response in the plant, which is initiated under mechanical stimulation to protect the tissue from damage.^[^ ^31^ ^]^

### 2.2 Plant Impedance Testing

**Figure 4:**
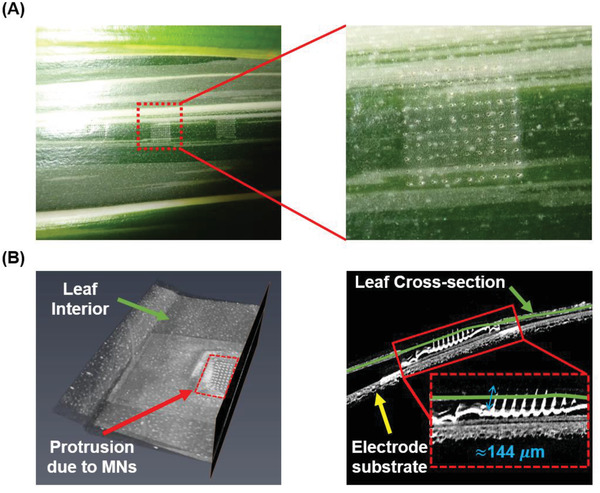
A) A top view image of the MNs penetration site after removal of the sensor showing the puncture marks. The inset shows a magnified image of the puncture site. B) 3D X‐ray cross‐sectional reconstruction image of the MNs while they are inserted into the leaf. The cross‐section is shown on the right, where the surface of the leaf can be seen to intersect the MNs. The penetration depth is shown in the figure inset.

To experimentally verify that the MNs can pierce the cuticle, the sensor is pressed against a freshly cut leaf of *Arabidopsis thaliana* and then promptly removed to inspect the site for signs of the piercing. As can be seen from **Figure** **4**a, a superficial inspection of the leaf under light microscopy shows puncture marks coinciding with the MNs array structure. This is confirmed by cross‐sectional X‐ray imaging of a leaf sample with the MNs inserted into the leaf (Figure 4b). The MNs appear to burrow into a depth of ≈144 µm into the leaf. The array is imaged again under light microscopy to validate their status (Figure S7, Supporting Information) and using an SEM (Figure S8, Supporting Information). Although the array may look intact macroscopically, upon detailed inspection with an SEM, the tips of 12% of the MNs in the array appear to be bent upon being dislodged from the leaf. This suggests that although the MNs may survive initial contact, dislodging can cause the tips to bend and thereby impair their utility for repeated usage.

The sensor is attached to an intact leaf in a plant growth chamber under controlled environmental conditions using 3M micropore tape (Surgical grade). The hypoallergic nature of the tape ensures that the attachment process does not result in the leaching of materials into the leaf after removal. Furthermore, the porous nature of the adhesive allows the leaf to be aerated. Two rounds of testing are conducted to characterize the sensors and assess their potential for long‐term deployment. For long‐term testing, the sensor terminals are connected to the working (WE), counter (CE), and reference (RE) electrode alligator clips of a portable potentiostat.

The plant specimens are exposed to a photoperiod (SciBrite light emitting diodes, Intensity of 350 μmoles m^−2^ s^−1^ with spectral peaks at 630, 520, and 450 nm) of 12 h at a temperature of 23 °C and 12 h of darkness at a temperature of 20 °C each day. The transition from light to darkness is abrupt as the hydroponic lights are turned off or on at full intensity during the cycling period. The soil is unchanged during the experimental test and the water level in the tray is maintained unless otherwise stated. The impedance of the tissue is acquired in situ from 10 Hz up to 100 kHz to probe the *α* and *β* dispersion regions with a 10 mV peak‐to‐peak sinusoidal stimulus. 360 frequencies are probed within the desired range and are spaced apart on a logarithmic scale. Prior to running each test, the open circuit potential is estimated with a stability tolerance of 0.01 mV s^−1^. The voltammetric test is thereby done with respect to the open circuit potential to aid in the reduction of the noise due to the plant's action potentials. During the test, the minimum sampling time, which is the period over which the stimulating potential was applied at each frequency, is set to 0.5 s. The impedance and phase shifts measured in the 0.5 s window are averaged and recorded.

**Figure 5:**
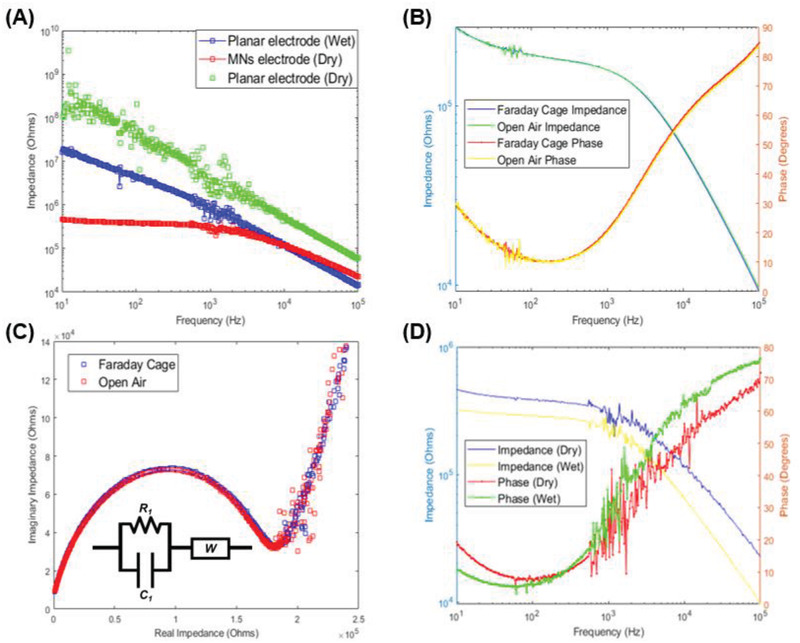
A) Impedance recorded for freshly cut leaf by the planar sensor with dry (green) and wet (blue) contacts and by MNs‐equipped sensor (red). B) Bode plots for impedance recorded in Faraday cage (blue line for impedance and red line for phase) and the open‐air (green line for impedance and yellow line for phase) by MNs‐equipped sensor. C) Nyquist plot constructed from impedance recorded in Faraday cage (blue) and the open‐air (red) by MNs‐equipped sensor. The equivalent circuit model fitted is shown as an inset in the plot. D) Bode plots for impedance recorded by MNs‐equipped sensor for plant specimen while hydrated (yellow line for impedance and green line for phase) and dry (blue line for impedance and red line for phase).

The first EIS test is conducted to compare the relative performance of the MNs‐equipped sensor to a conventional planar sensor on the same site (**Figure** **5**a). The planar sensor is subsequently coated with an electrolyte gel (GEL100, BIOPAC Systems, Inc.) to repeat the test and compare the effect of the dry and wet contacts on the impedance spectrum. Between 10 Hz and 1 kHz, the MNs‐equipped sensor reports impedance values that are approximately one and two orders of magnitudes lower than the planar sensor under wet and dry contact settings, respectively. This indicates that the MNs can establish direct contact with the ion‐rich layers of the leaf that lie under the cuticle thereby reducing the resistance detected by the sensor. This is confirmed by the equivalent circuit models that show that the interfacial layer of resistance and capacitance of the cuticle is effectively shorted (Figure S9, Supporting Information). This frequency range is of valuable analytical value for the assessment of the physiological state of the plant tissue as it lies squarely in the *α* dispersion region. Dispersion in this region occurs due counter ion polarizations according to the colloidal particle mechanism, which is affected by the membrane permeability of the tissue. Although the impedance in this band is relevant to the physiological state of plant tissue, true measurements in this range remain difficult due to the complicated tissue composition and polarization effects.^[^ ^32^ ^]^ The use of MNs‐equipped sensors may aid in the further exploration of this dispersion region and thereby in the true assessment of the physiological state of the plant. At frequencies higher than 1 kHz, the MNs‐equipped sensor and planar sensor under wet contact setting converge to similar values of impedance. This is to be expected as at high frequencies the cuticle is effectively shorted electrically and has no marked impact on the impedance. However, the MNs‐equipped sensor reports impedance values that are still lower than the planar sensor under dry contact settings. Uniquely, the MNs‐equipped sensor displays a higher aversion to motion artifacts due to the swaying of the leaf than the planar sensor under both contact settings, which is reflected in the lower noise magnitude in the impedance spectrum acquired by the MNs‐equipped sensor under simulated windy conditions (Figure S10, Supporting Information).

To evaluate the sensitivity of the EIS measurement to interference, the test is repeated using the MNs‐equipped sensor in open‐air conditions and inside a Faraday cage (Figure 5b). The effects of electromagnetic interference can be seen in the Bode plot, with the phase shift being more sensitive to noise than the raw impedance value. However, the noise level is still not sufficient to distort the acquired signal significantly as seen in the Nyquist plot (Figure 5c) resulting in 2.3%, 8.5%, and 10% deviations between the fitted resistive, capacitive, and Warburg circuit elements, respectively, for the Faraday cage and open‐air tests. The relative noise in measured impedance and phase shift values are calculated using the impedance spectra and phase shifts acquired in the Faraday cage as the reference (Figure S11, Supporting Information). During acquisition, the built‐in notch filter of the potentiostat is used to attenuate the Mains noise. As the cables connecting the sensor to the potentiostat are unshielded, this leaves them susceptible to electrical interference due to proximity to other electrical equipment. The relative noise in the impedance is found to range between a high of 9% around 58 Hz and a low of 0.1% at 1.18 kHz. Similarly, the relative noise in the phase shift is found to range between a high of 50% around 50 Hz and a low of 0.3% at 206 Hz. The average relative impedance and phase shift noise values across several specimens (Figure S12, Supporting Information) were found to be 3.83% and 7.61%, respectively. This suggests that it may be feasible to deploy the sensor in open‐air farms by probing frequencies that are less susceptible to noise and acquiring the bioimpedance from these frequencies. The choice of probed frequency may depend on both the measuring instruments and the tissue. Furthermore, previous literature shows that changes in the hydration of the tissue can be correlated to changes in the measured impedance.^[^ ^33^ ^]^ To detect this effect using the MNs‐equipped sensor, an *Arabidopsis thaliana* leaf is cut and dried for several hours. The impedance of the dry sample is measured and compared to its initial impedance at the onset of the experiment (Figure 5d). The sensor reports an increase in the impedance values upon loss of hydration in the leaf, which is consistent with what was reported previously.

Previously, we demonstrated that analyzing the impedance spectrum of Barley leaves (*Hordeum vulgare*) has shown that the impedance at 10 Hz cycles in a diurnal fashion entrained by light.^[^ ^10^ ^]^ Although the physiological phenomena explaining this cycling pattern are unclear, it may serve as a useful tool to assess light exposure of crops in a field, which is crucial for photosynthetic activities. As such, a specimen of *Arabidopsis thaliana* is monitored using the MNs‐equipped sensor for 12 d to discern if there is a cycling pattern that is also entrained by light similar to that of Barley. This experiment also serves to illustrate the long‐term effects of the sensor attachment to the leaf as it has been reported previously that plants tend to seal epidermal wounds to maintain turgor pressure in the leaf,^[^ ^34^ ^]^ which could impact the impedance measured overtime. The plants were regularly watered to maintain a constant level of water in the growth tray.

**Figure 6:**
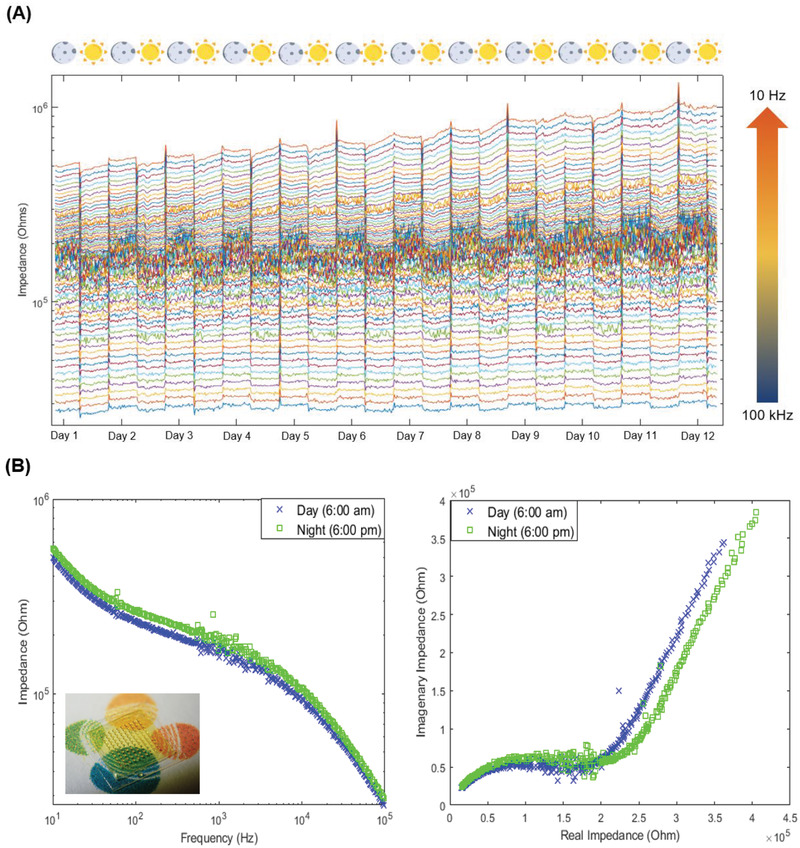
A) Magnitude of electrical impedance recorded over time for frequencies between 10 Hz and 100 kHz. The frequencies are plotted in descending order from bottom to top as the impedance is higher at low frequencies. B) The Bode (left) and Nyquist (right) plots comparing the spectra of the specimen in the day (blue cross) and night (green square). An inset shows the polyimide MN array used for the measurement.

**Figure 7:**
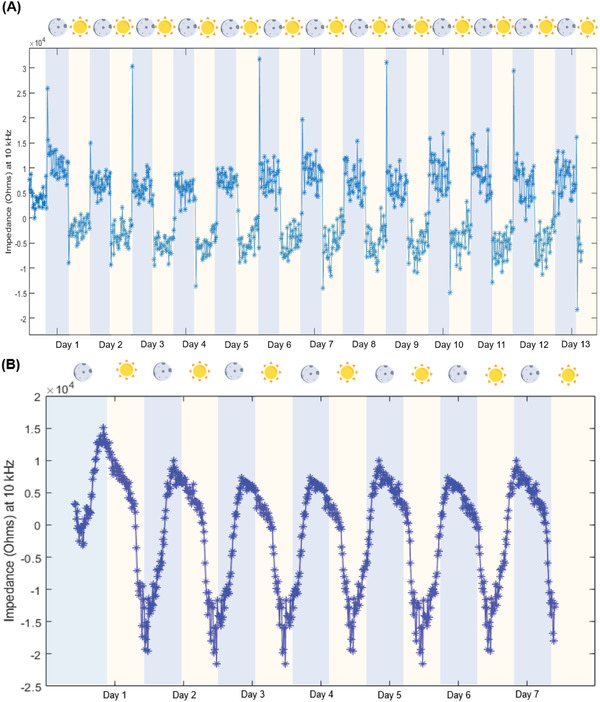
A) The impedance values at a frequency of 10 kHz plotted over 13 d for an *Arabidopsis thaliana* specimen. The shading indicates the lighting condition, where the yellow shade is for the day and the blue shade is for the dark. B) The impedance values at a frequency of 10 kHz plotted over 7 d for an *Arabidopsis thaliana* specimen under natural light. The shading indicates the lighting condition, where the yellow shade is for the day and the blue shade is for the dark.

As can be seen in **Figure** **6**a, there is a discernable cycling of impedance at the various frequencies probed in response to light. The impedance values tend to rise once the photoperiod is over and fall once it starts. The Bode and Nyquist plots for the specimen in the morning compared to the night (Figure 6b) show that the impedance value at each frequency is higher in the night than in the day. The cycling behavior is clearest when probing the impedance value at 10 kHz over time (**Figure** **7**a). During the transition from light to dark, the impedance value rises abruptly before leveling down to a value that is still higher than that of the day photoperiod. Planar electrodes failed to detect this cycling behavior (Figure S13, Supporting Information). The swaying of the leaf causes spikes in the impedance recorded by the planar electrodes as they slide over the surface of the leaf. This further highlights the importance of using MNs for stable contact and sampling the EF.

The leaf is observed for any signs of discoloration or senescence which may affect the measured impedance during and after the experiment. It is difficult to observe the puncture site directly during data acquisition as it was obscured by the sensor and the micropore tape. Removal of the sensor for inspection may induce mechanical stimuli that would affect the recorded impedance. To ensure that the wounds caused by the MNs puncturing the leaf do not lead to permanent structural damage in the tissue, a separate specimen was subjected to an identical puncturing wound by the MNs after which they were subsequently removed. The specimen was housed in a green‐house environment to control for environmental conditions and to protect it from fungal pathogens that may use the puncture site to facilitate infestation. The wound healing response was studied over time by imaging the site of the puncture to monitor the sealing of the site (Figure S14, Supporting Information). By the 4th day postwounding, the puncture site is occluded and the microholes are sealed. The same response was observed for the specimens used for long‐term impedance monitoring. This robust wound response indicates that the application of the sensor on the leaf has not caused any long‐lasting detrimental effects on the integrity of the tissue.

**Figure 8:**
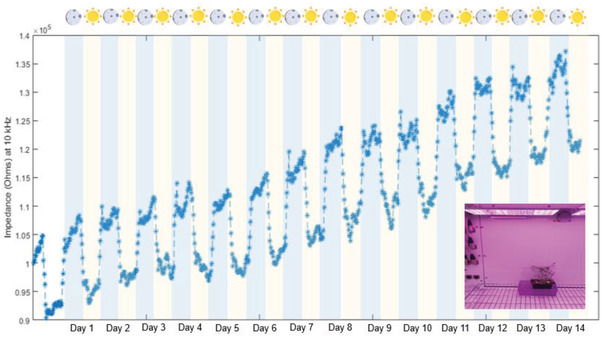
The impedance values at a frequency of 10 kHz plotted over 14 d for the same specimen while undergoing progressive drying. An inset shows the specimen in the growth chamber whilst undergoing drying.

This trend in impedance may be attributed to the sharp transition between light and dark in the growth chamber. To see if this trend is preserved under natural light, the experiment is repeated under natural lighting conditions. The plants are placed in a greenhouse to control environmental conditions, such as temperature and humidity, but were exposed to natural lighting. The impedance spectra are acquired using the same methodology applied in the growth chamber. The impedance at 10 kHz over time displays the same periodic trend observed in the growth chamber (Figure 7b). However, the rise and descent in the impedance are now gradual further corroborating that this is entrained by light. The cyclical nature of the impedance may be related to the initiation of photosynthetic activity, which allows the leaf to act as a sink for water osmosis, thereby lowering the impedance of the tissue. To assess this theory, the watering of the specimen was halted, and the impedance was monitored over time (**Figure** **8**). The impedance value at 10 kHz steadily rose over time as compared to the standard baseline previously established for the specimen suggesting that this may indeed be related to water transport in the leaf. Moreover, the average difference between the peak and the trough of the cycling impedance at 10 kHz increased steadily under dehydration. Potentially, this can be used to indirectly assess photosynthetic activity in the tissue as the two phenomena are interlinked without the need for sensitive biopotential measurements as was explored by Lautner et al.^[^ ^35^ ^]^

## 3 Conclusion

In summary, a novel micromolding and release strategy has been demonstrated for high fidelity replication of MNs for the fabrication of impedimetric plant sensors. The utility of the method is demonstrated via the reproduction of MNs composed of either photocurable or thermosetting polymers. Using a swelling‐based release technique, the need for applying lateral bending forces to demold is negated. This expands both the scope of possible designs for replication and the choice of materials used in micromolding. The robustness of this method allows for the reuse of the elastomeric mold for upward of 20 replication cycles with little to no deformation in the released geometry. Integrating the MNs in plant sensors can allow for direct sampling and assessment of the EFs of plant tissue, which is crucial to obtain accurate and sensitive data regarding the health status of crops. Deployment of such sensors did not compromise the leaf's wound healing response as the puncture site is occluded shortly after the removal of the MNs. MNs‐equipped sensors demonstrably outperform conventional planar sensors in plants via more sensitive reporting of impedance values, thereby detecting discernable changes in the physiological state of the probed plant tissue. There is also a potential for open‐air deployment of such sensors as they benefit from being averse to motion artifact induced noise. Similarly, the effect of electromagnetic interference on the impedance spectra can be minimized by exclusive probing of frequencies less susceptible to noise without the need to resort to heavy shielding of wires.

An important advantage of the developed micromolding method is that it can be used to fabricate 3D electrodes with a wide range of possible designs and high resolution, due to the utilization of TPP 3D printing for making the master. At the same time, this method allows for reproducible and scalable fabrication without compromising on fidelity, rendering it a feasible solution for many sensor fabrication applications.

## 4 Experimental Section

### Master Mold Fabrication

The master mold computer‐aided design (CAD) file was designed using AutoCAD software suite (AutoDesk) to incorporate a 10 by 10 array of conical MNs with a radius of 70 µm and height of 350 µm and a spacing of 300 µm on a flat rectangular 2 by 2 mm^2^ bed that is 100 µm thick. The CAD file was converted to a general writing language (GWL) file using Describe software (Nanoscribe, GmbH). The software splits the structure in the horizontal and vertical axes to configure the exposure path of the laser. The vertical and horizontal distances between the slices are referred to as the slicing and hatching distances, respectively. Both the slicing distance and hatching distance were set to 0.5 µm. A shell and scaffold configuration was chosen to accelerate the TPP‐direct laser lithography process. The outermost layer of the structure is referred to as the shell and the lines demarcating its slices are fully exposed by the laser. The thickness of the shell was chosen to be 10 µm. The interior of the structure was filled with a triangular scaffold, which was also hatched and sliced with distances of 0.5 µm. The writing field and piezoelectric motor used to articulate the laser in the instrument limits the voxel size to 300 by 300 by 300 µm^3^. A secondary galvo motor is engaged to move the laser between each voxel, which necessitates stitching of the voxels. To ensure adhesion of the individual voxel, a block overlap distance of 2 µm is used. At the interface of the voxels, a stitching line can be seen due to the minute misalignments during movement of the stage and exposure. A 25 by 25 mm^2^ single‐sided polished Silicon die was sequentially cleaned in acetone, isopropanol, and DI water. IP‐S resin was decanted into the center of the die, which was subsequently loaded into the Photonic Professional GT system (Nanoscribe, GmbH). The exposure process of the master template was completed in 2 h and 35 min.

Upon completion of the polymerization process, the die was submerged in mer‐Dev 600 (MicroChem) to remove the uncured resin and then washed in isopropanol.

### PDMS Embossing

An acrylic rectangular 20 by 20 mm^2^ frame was cut using a CO_2_ laser. The frame was placed around the master mold and attached to the Silicon die using drops of chloroform ejected from a hypodermic needle nozzle. PDMS (Sylgard 184) was mixed in a 10:1 ratio (monomer to curing agent) vigorously and then outgassed for 45 min. The PDMS was then cast on the master mold and the excess was removed with a surgical blade. The mold was then outgassed for an additional 20 min and then moved to a convection oven set at 90 °C where the PDMS was cured for 30 min. To remove the replica from the master mold, a surgical blade was used to trim the edges and release the PDMS.

### SU‐8 Micromolding

150 µL of SU‐8 2005 (solid content of 45%, a viscosity of 45 cSt, and a density of 1.164 g per mL) was cast onto the PDMS mold under yellow light using a transfer pipette. The mold was outgassed for 20–30 min to ensure adequate flow of the varnish into the cavities. The mold was fixed in a glass holder at ambient temperature. The holder was moved at a speed of 2.5 mm s^−1^ under a stationary blade fixed perpendicular to the surface of the mold. As the mold passed under the blade, the varnish was spread on the surface of the mold. A surgical blade was used to remove the excess around the mold cavity. The resulting thickness was characterized using a profilometer after the MNs were released. The mold was then soft‐baked at 65 °C for 3 min on a hot plate. The temperature was then ramped at a speed of 3° C min^−1^ to 95 °C and was held at that point for 5 min. Depending on the size of the design of the master mold, evaporation of the SU‐8 may cause significant shrinkage and may necessitate a supplementary addition of another layer of SU‐8 on top, which is then soft‐baked. This may be repeated until the dry SU‐8 fills the mold cavities. The mold was then exposed to UV light (365 nm) with the exposure dose depending on the thickness of the design. It was found that for designs that were 500, 300, and 100 µm thick, exposure doses of 600, 350, and 150 mJ cm^−2^ were adequate, respectively. If the design exceeds 500 µm in thickness then a dual exposure is necessary, where the first exposure round exposes the upper surface of the mold and the second round exposing through the PDMS surface at the bottom of the mold. Finally, the mold was hard‐baked at 95 °C for 30 min to crosslink SU‐8 and was subsequently cooled to room temperature at a ramp speed of 10 °C min^−1^.

### Polyimide Micromolding

Polyimide varnish (PI‐2611) (solid content of 13%, a viscosity of 13 500 cSt, and a density of 1.082 g per mL) was cast onto the PDMS mold and was outgassed for 20–30 min to allow the varnish to seep in completely into the cavities of the mold. The same blade coating technique used for SU‐8 was also used to spread the polyimide varnish on the mold. The excess varnish around the mold cavity was removed using a surgical blade. The mold was then baked at 90 °C on a hot plate for 1 min and 30 s and then on 150 °C for 1 min and 30 s. The mold was then allowed to return to room temperature and was then hard baked from 23C to 250 °C at a ramp speed of 4 °C min^−1^. The mold was held at 250 °C for 30 min to complete the imidization process and was subsequently cooled gradually to return to room temperature at a ramp speed of 10 °C min^−1^.

### Mold Release

The mold was submerged into chloroform in a glass beaker (80 mL). The beaker was sealed to prevent evaporation of the solution and minimize toxic fume release. Inside a chemical fume hood, the beaker was placed in a water bath, which was ultrasonicated for 10–15 min at a frequency of 76 Hz. The MNs array was gradually deflected out of the PDMS matrix. Depending on the size of the design, a surgical blade may be needed to tip the sides of the array to allow chloroform to seep into the cavities. Finally, the mold was submerged in a solution of 50% ethanol in DI water to restore its previous shape and the molded polymer could be cleaned in isopropanol to remove any residual chloroform.

## Conflict of Interest

The authors declare no conflict of interest.

## Supporting information

Supporting Information

## Data Availability

Research data are not shared.
