# Supplementary material for: Robust, Long‐Term, and Exceptionally Sensitive Microneedle‐Based Bioimpedance Sensor for Precision Farming
Source: Adv Sci (Weinh). 2021 Jun 17;8(16):2101261. doi: 10.1002/advs.202101261 (PMC8373106; doi:10.1002/advs.202101261)
Supplement: Supplementary file 1 — Supporting Information [file ADVS-8-2101261-s001.pdf]

© 2021 Wiley-VCH GmbH

ADVANCED SCIENCE Open Access

Supporting Information

for *Adv. Sci.*, DOI: 10.1002/advs.202101261

Robust, Long-term and Exceptionally Sensitive Microneedle-based Bioimpedance Sensor for Precision Farming

Abdullah Bukhamsin, Khalil Moussi, Ran Tao, Gilles Lubineau, Ikram Blilou, Khaled Nabil Salama, and Jürgen Kosel

© 2021 Wiley-VCH GmbH

Supporting Information

Robust, Long-term and Exceptionally Sensitive Microneedle-based Bioimpedance Sensor for Precision Farming

Abdullah Bukhamsin, Khalil Moussi, Ran Tao, Gilles Lubineau, Ikram Blilou, Khaled Nabil Salama, and Jürgen Kosel

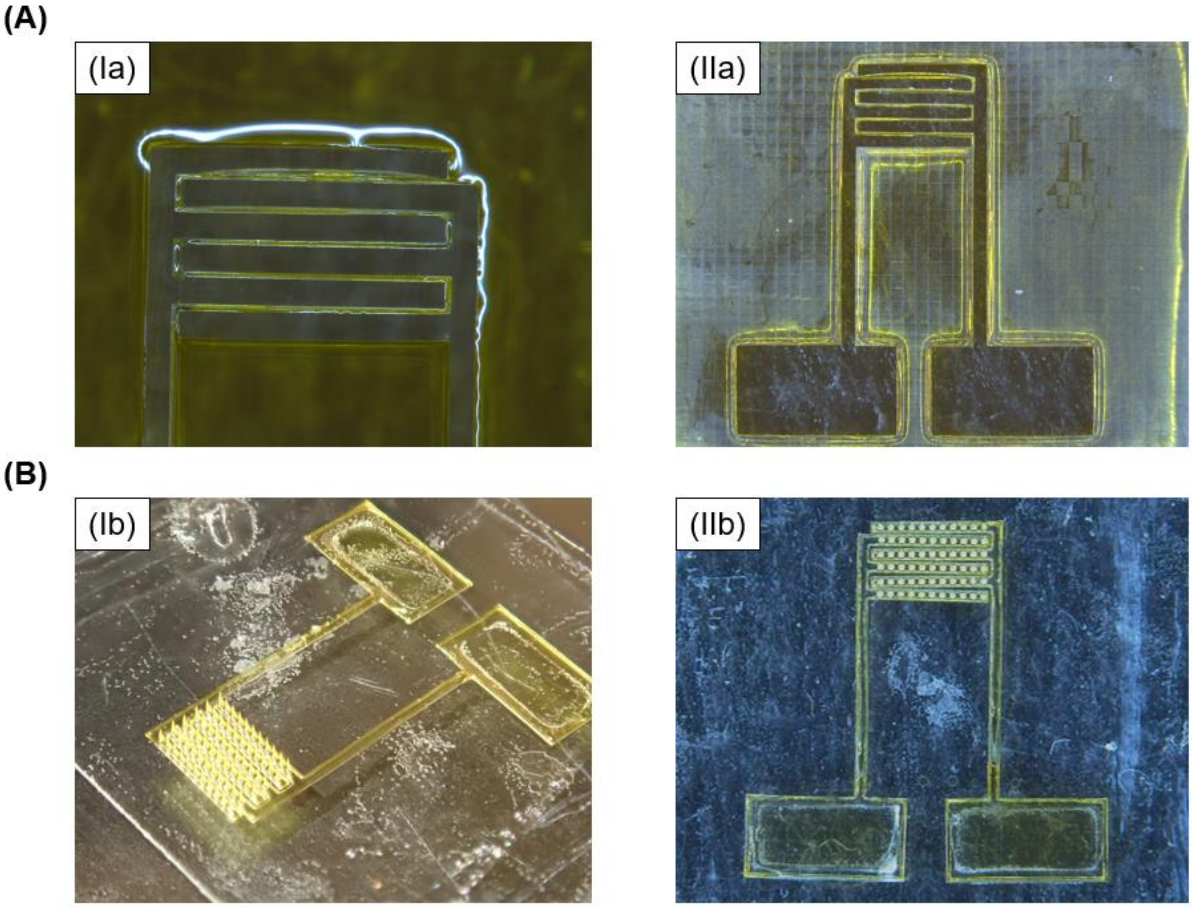

Figure S1. (A) Polyimide shadow mask with hollow digits 300 µm high and 50 µm in spacing fabricated using the reported micromolding and release strategy. **(B)** Interdigitated array with a spacing of 50 µm decorated with 300 µm high polyimide MNs for potential electrochemical sensing applications.

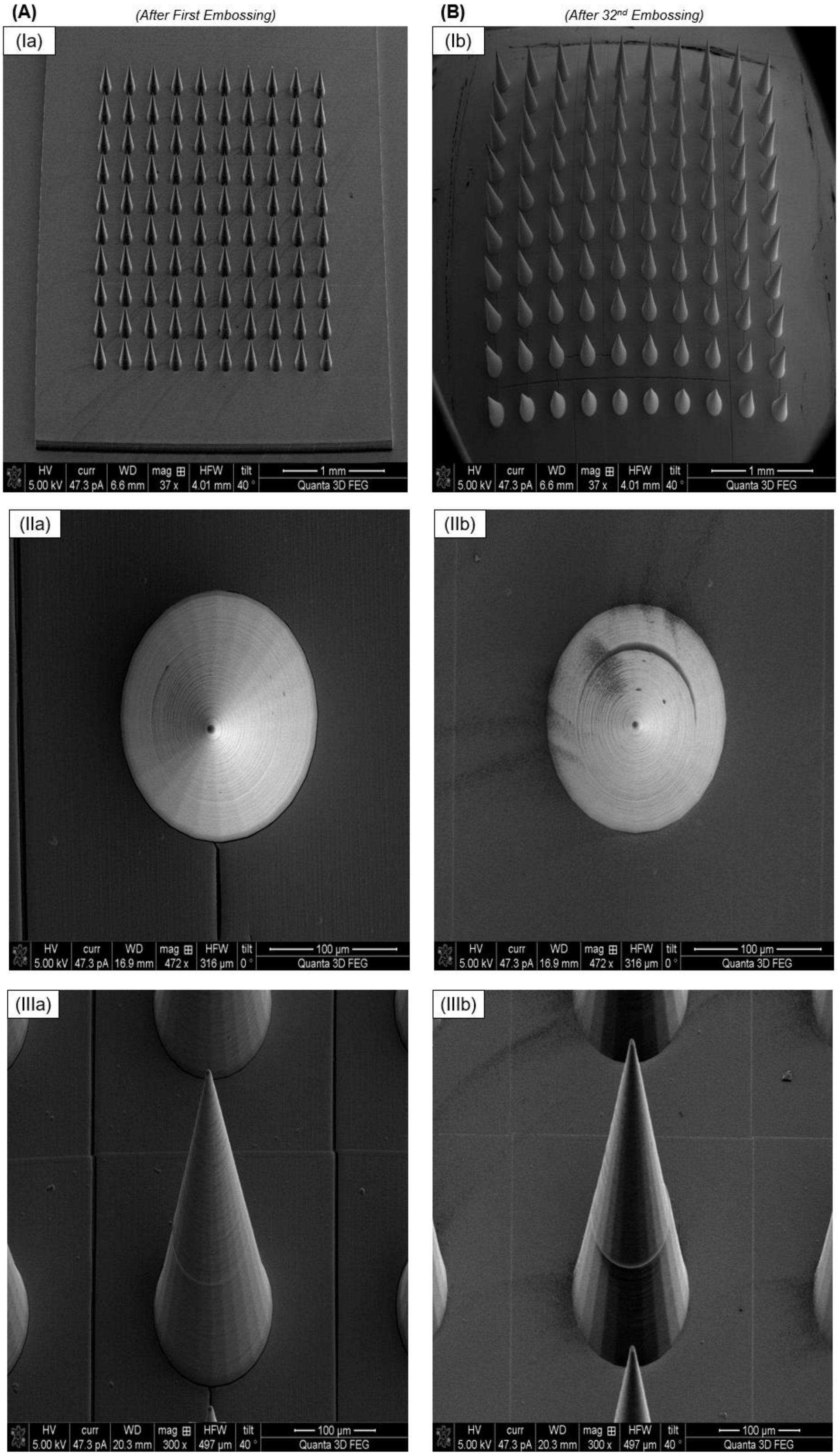

Figure S2. (A) SEM images of the master template after the first replication cycle from the top **(Ia)** and showing an individual MN from the top **(IIa)** and at an angle of 40° **(IIIa) (B)** SEM images of the master template after the 32nd replication cycle from the top **(Ib)** and showing an individual MN from the top **(IIb)** and at an angle of 40° **(IIIb).**

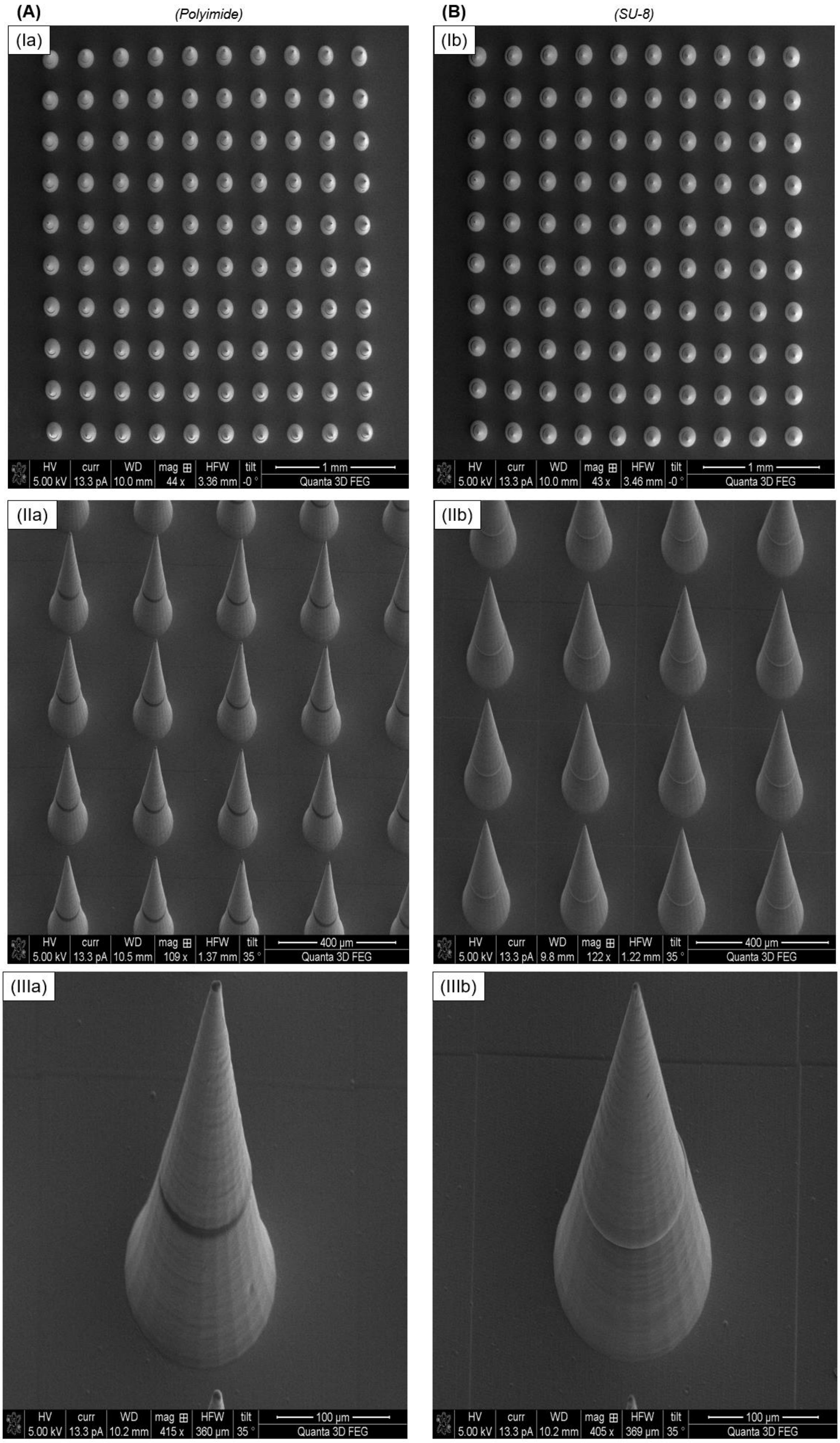

Figure S3. (A) SEM images of polyimide MNs from a top view **(Ia)**, isometric view **(IIa)**, and a single MN **(IIIa)**. **(B)** SEM images of SU-8 MNs from a top view **(Ib)**, isometric view **(IIb)**, and a single MN **(IIIb)**. The MNs arrays shown were fabricated from the same elastomeric mold.

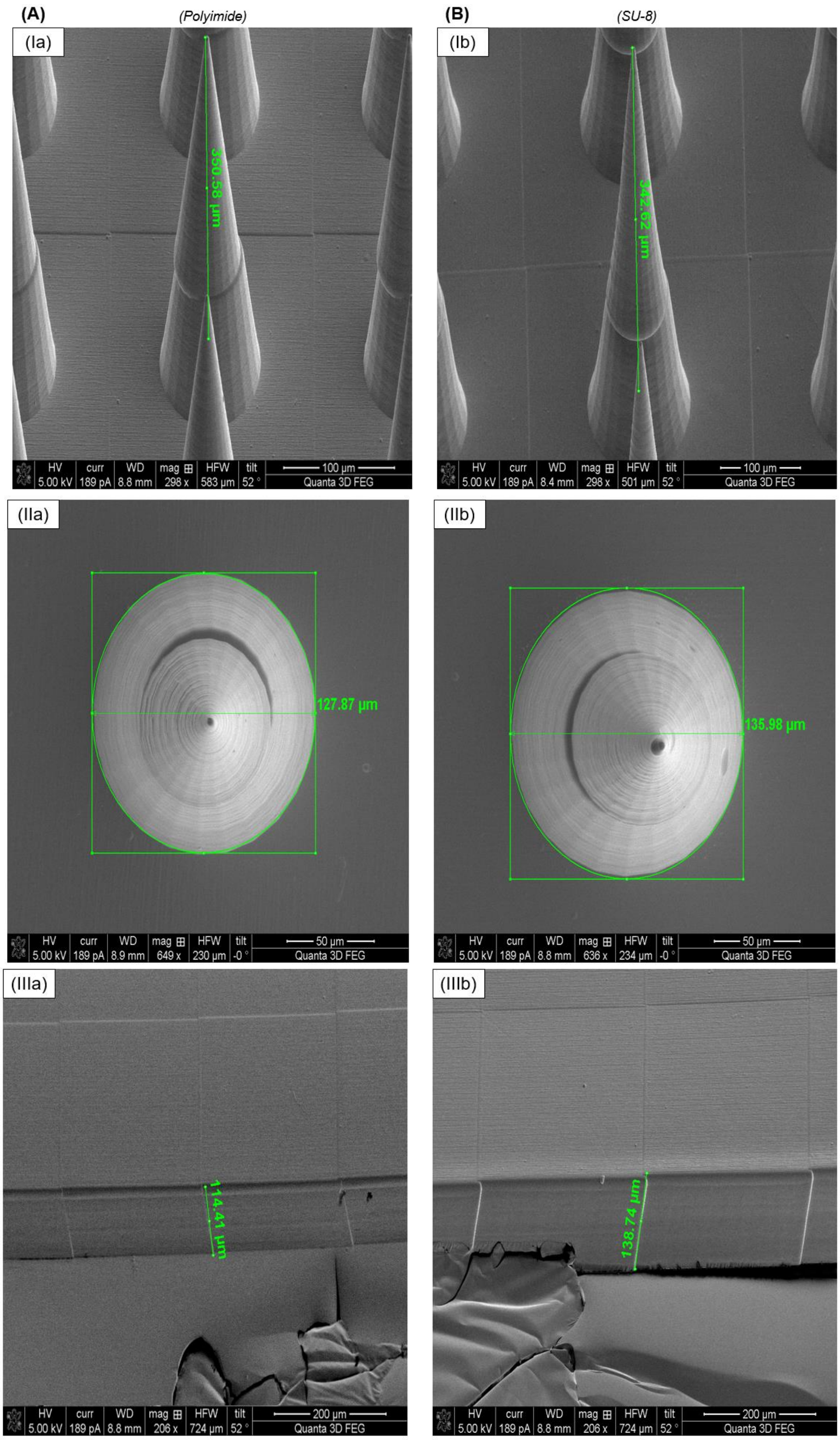

Figure S4. (A) SEM images of polyimide MNs with tilt-corrected measurements showing shank height **(Ia)**, base radius **(IIa)**, and bed thickness **(IIIa)**. **(B)** SEM images of SU-8 MNs with tilt-corrected measurements showing shank height **(Ib)**, base radius **(IIb)**, and bed thickness **(IIIb)**.

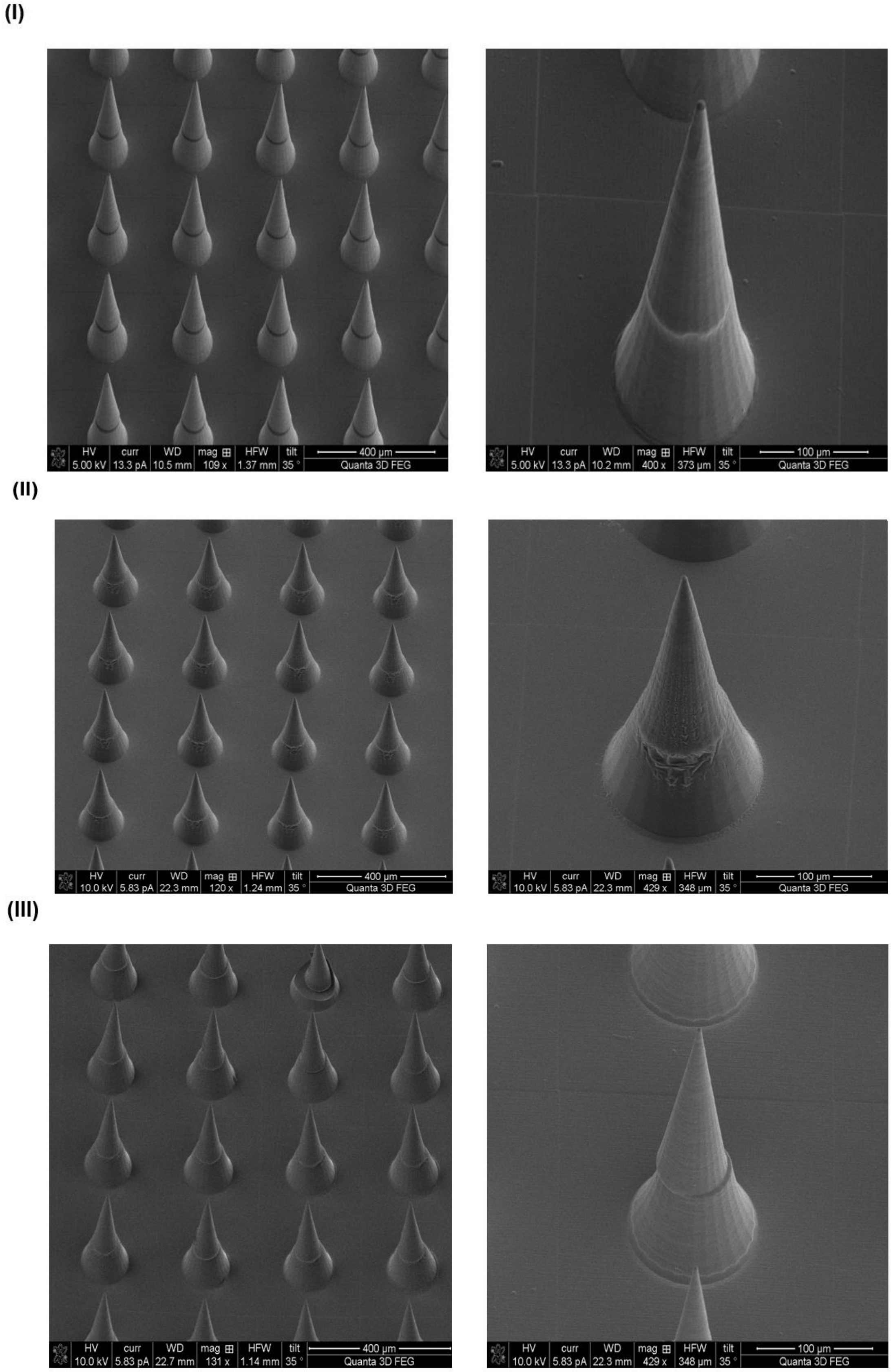

Figure S5. SEM images of polyimide MNs demolded from the same intermediary elastomeric master mold at the first **(I),** tenth **(II),** twenty-fifth **(III)** replication cycles, respectively.

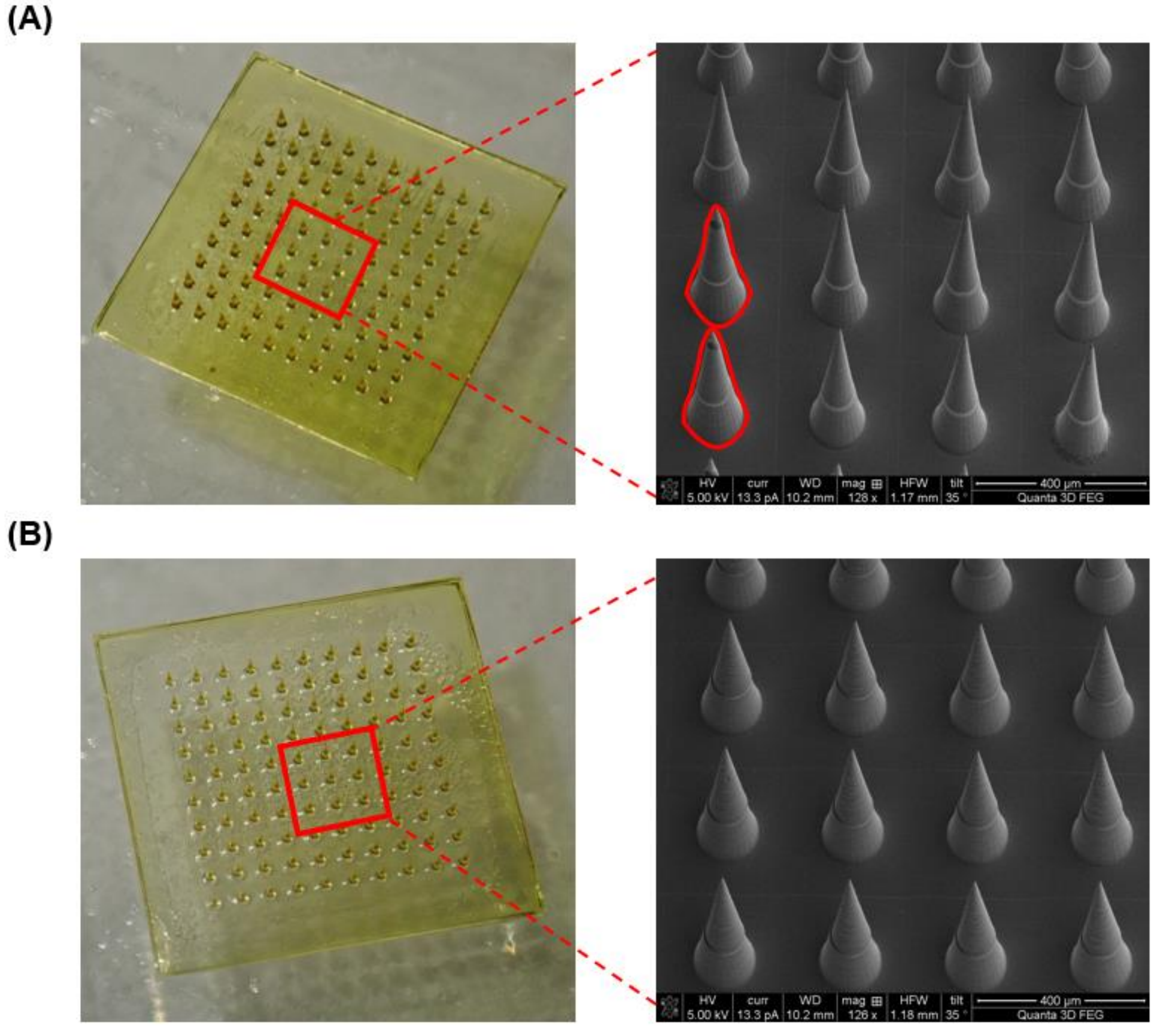

Figure S6. (A) Polyimide MNs released with bent tips imaged using a light microscope at 4x magnification. The inset shows the bent tips in an SEM graph with the deformed MNs highlighted in red. **(B)** A reference polyimide MN array with the desired geometry of the tips with an inset showing the tips in an SEM graph.

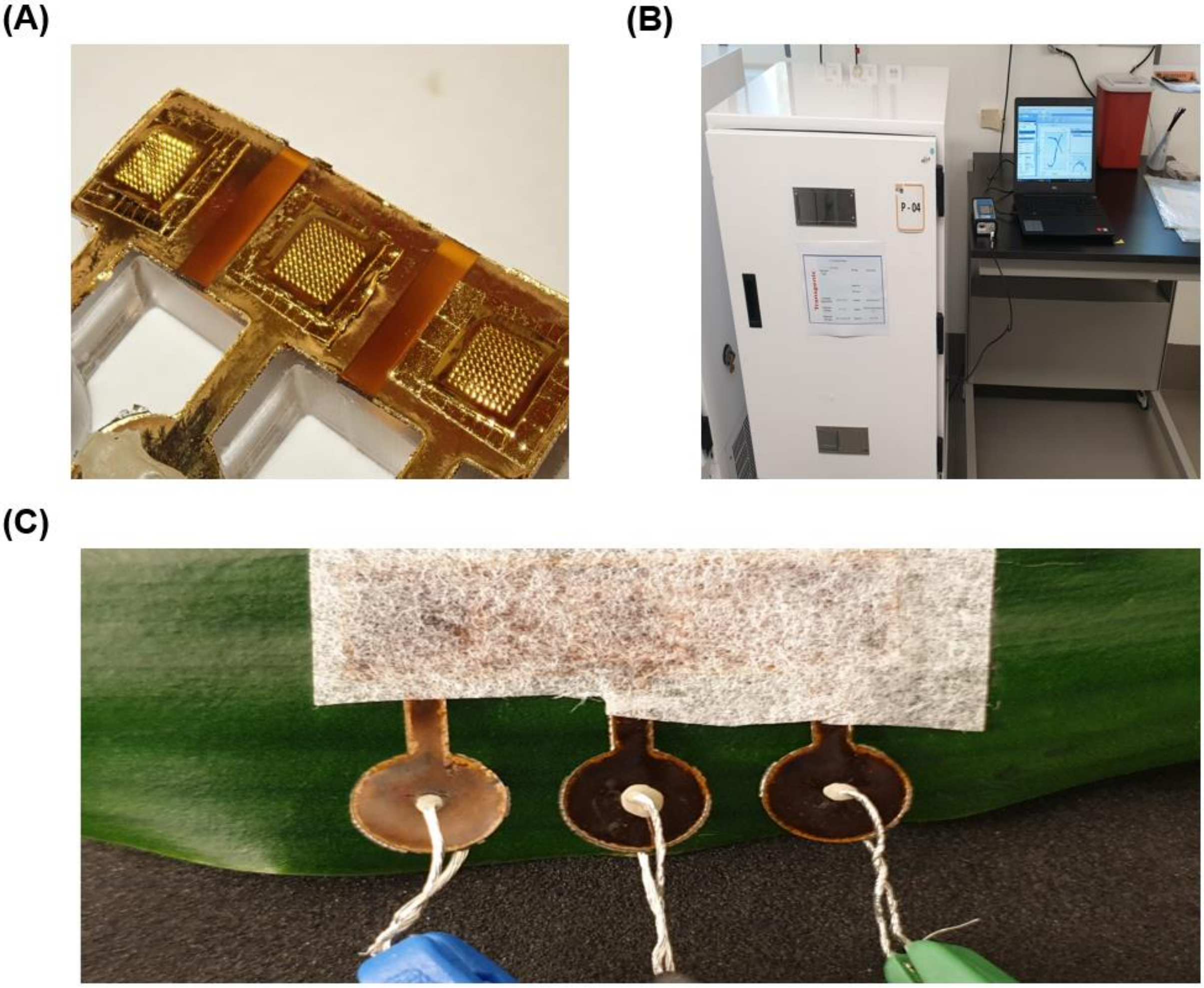

Figure S7. **(A)** An isometric view of the MNs on the sensor after removal from the leaf. **(B)** An image showing the plant growth chamber setup that was used to acquire the impedance data and to control for environmental conditions. **(C)** A close-up image of the MNs-equipped sensor as it is attached to the leaf.

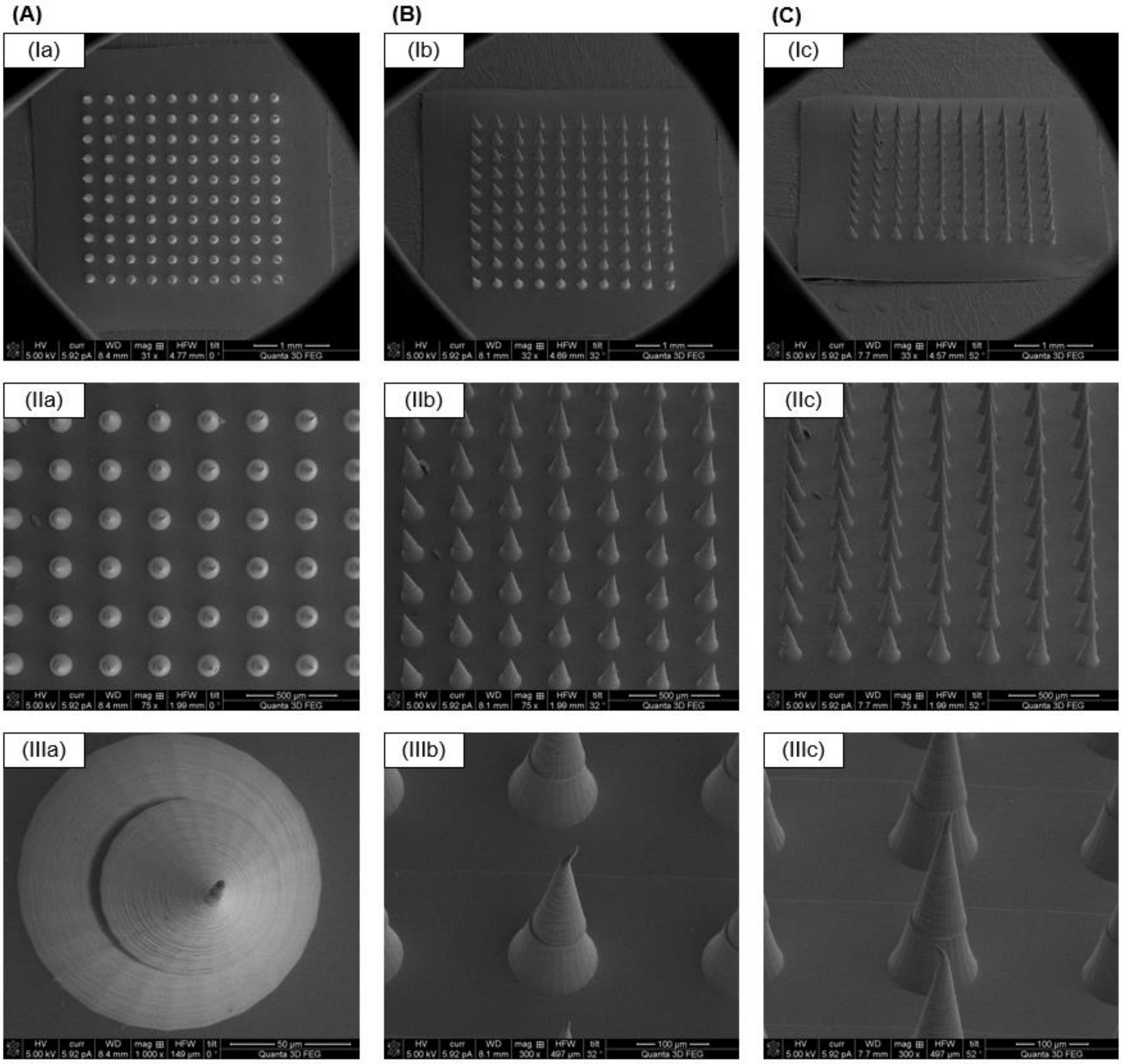

Figure S8. SEM micrographs of the MNs after puncturing the surface of the plant leaf. The micrographs depict the array from the top **(A)** at several magnification levels and an angle of 32° **(B)** and 52° degrees **(C).**

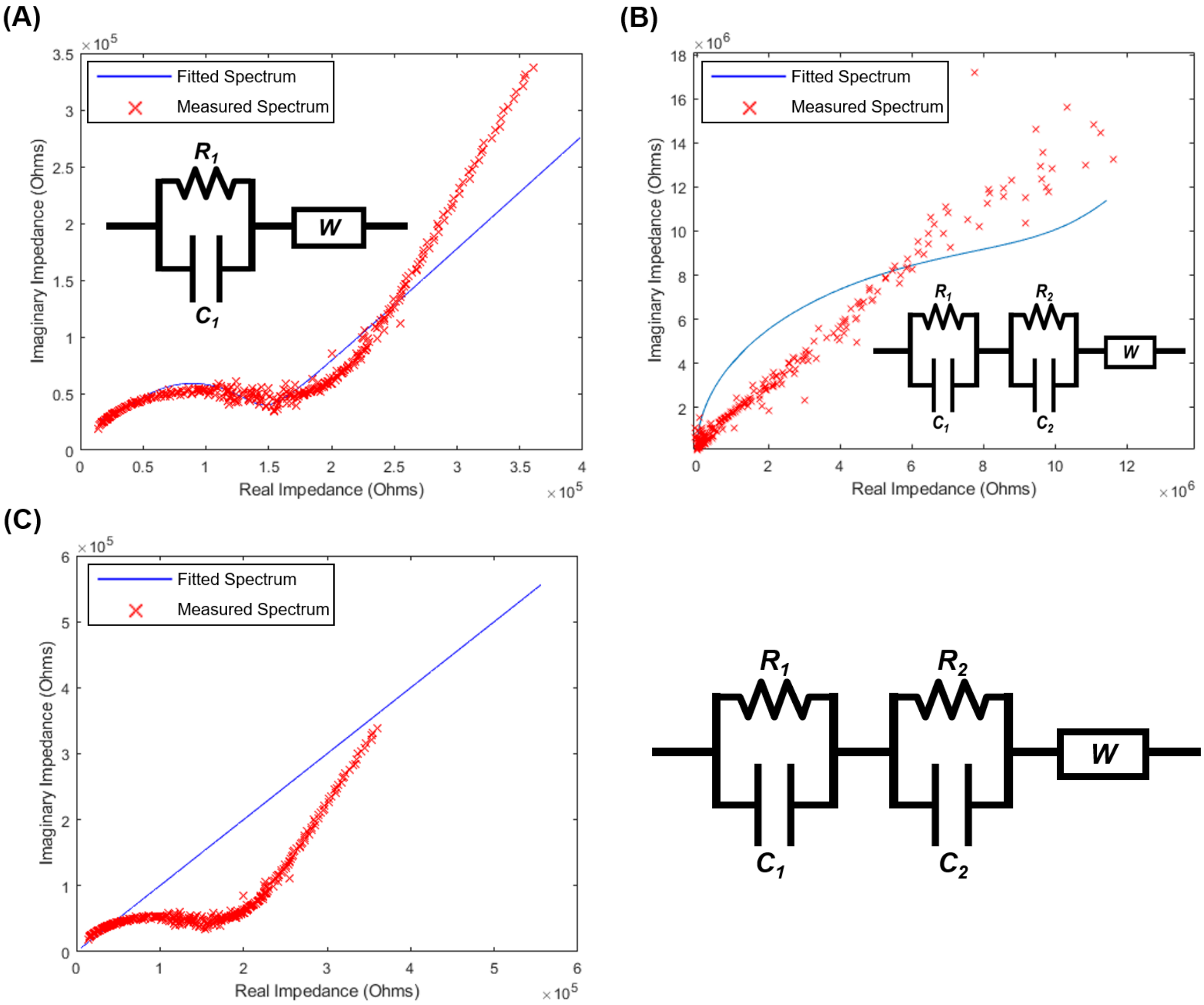

Figure S9. (A) Equivalent circuit model fitted on EIS measurements of MNs-equipped sensor on a wet leaf. The values of R_1, C_1, and W_A coefficient were determined to be 95 kOhm, 7.9 pF, and $2.6*10^{-6}$ Ohms per $\sqrt{}$ second, respectively. **(B)** Equivalent circuit model fitted on EIS measurements of planar electrodes on a wet leaf. The values of R_1, R_2, C_1, $C_{2,}$ and W_A coefficient were determined to be 36 kOhm, 193 kOhm, 10 nF, 43 nF, and 7.02 Ohms per $\sqrt{}$ seconds, respectively **(C)** Attempt to fit equivalent circuit model of planar electrodes on impedance spectrum measured using MNs-equipped sensor.

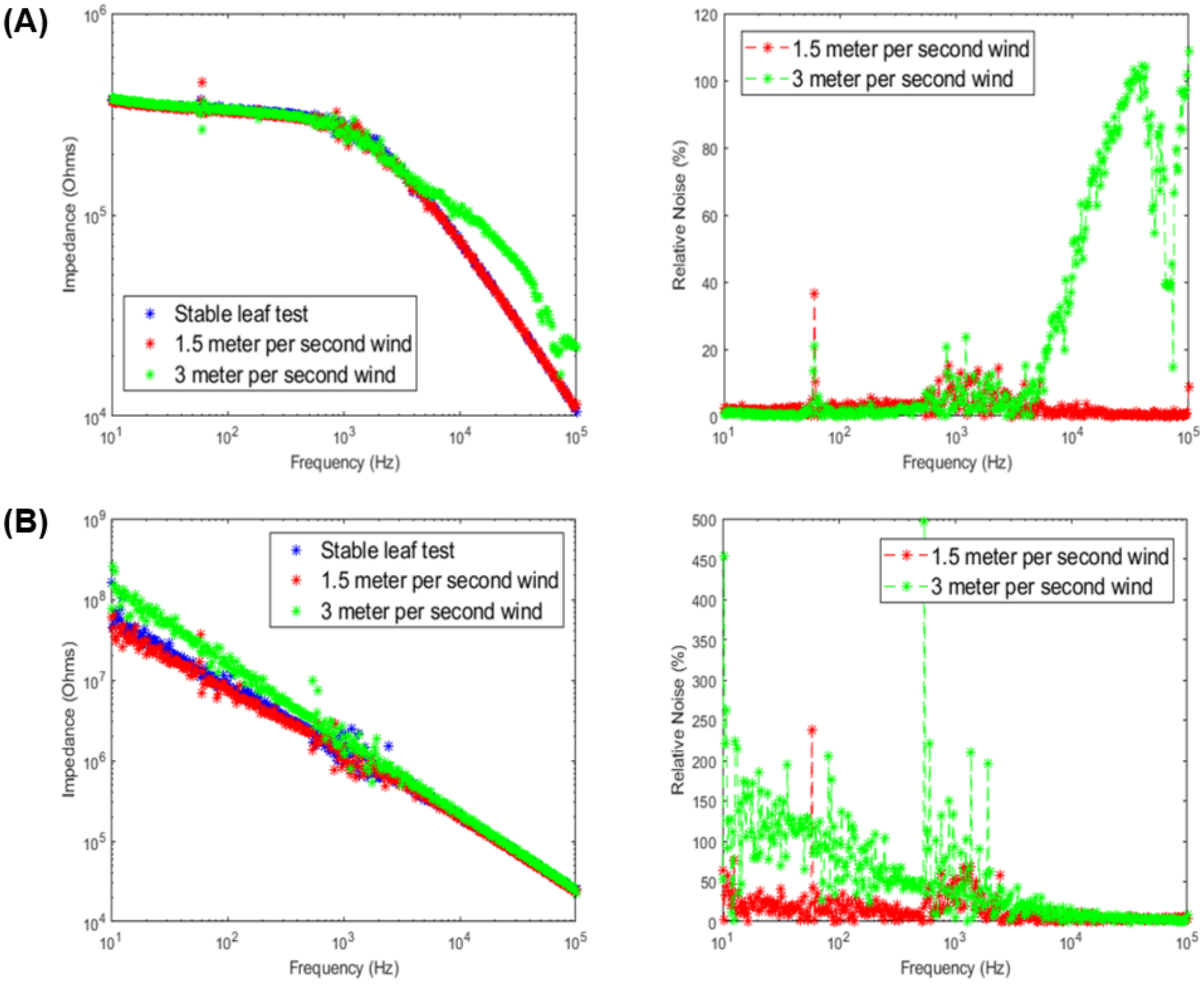

Figure S10. (A) Bode plots of MNs-equipped sensors showing their performance under static conditions (blue stars) relative to simulated windy conditions of 1.5 meters per second (red stars) and 3 meters per second (green stars). The relative error in the measurements obtained under simulated wind is plotted with reference to the static conditions over the frequencies probed. **(B)** Bode plots of planar sensors showing their performance under static conditions (blue stars) relative to simulated windy conditions of 1.5 meters per second (red stars) and 3 meters per second (green stars). The relative error in the measurements obtained under simulated wind is plotted with reference to the static conditions over the frequencies probed.

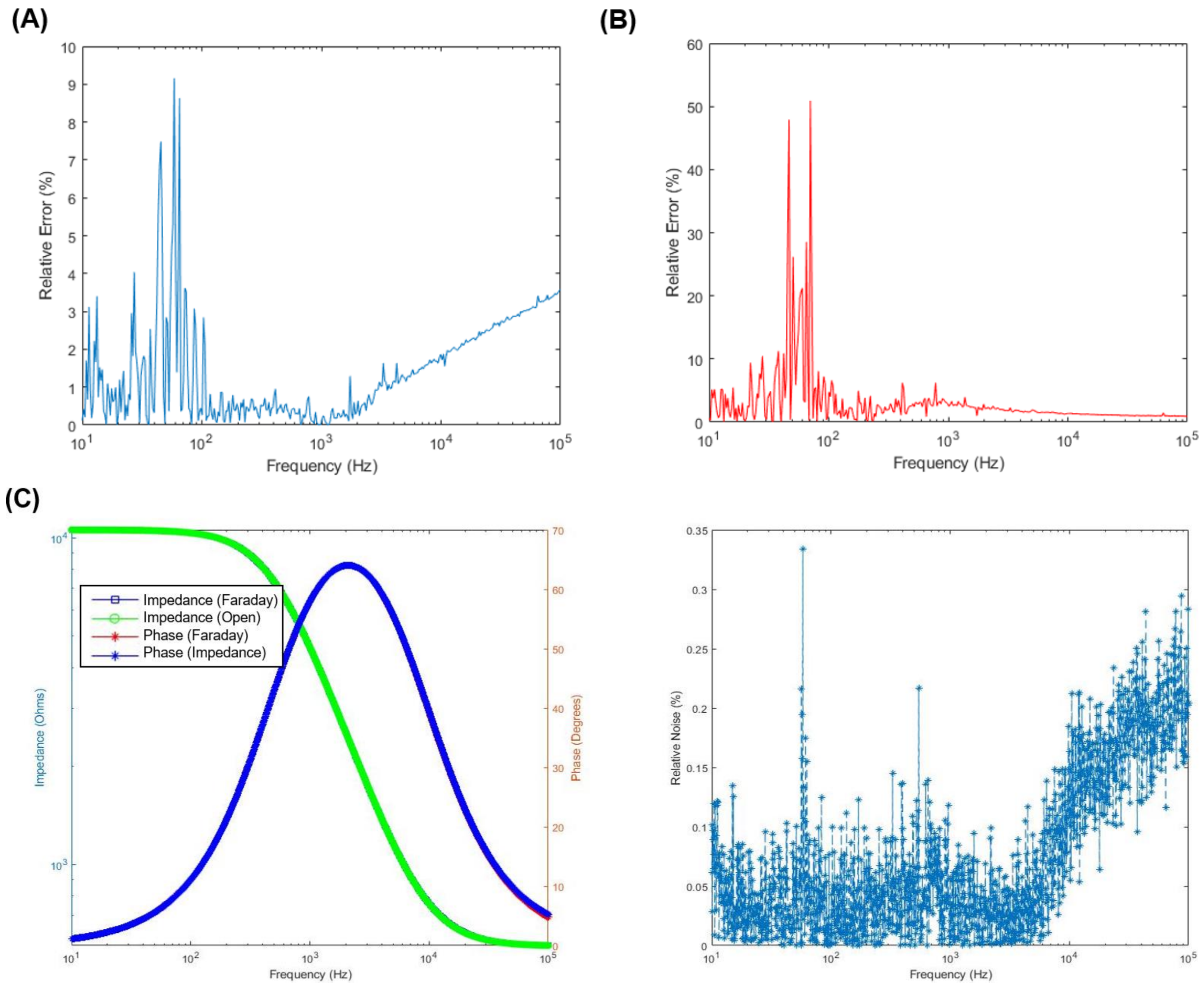

Figure S11. (A) Relative noise in impedance is plotted as a function of frequency. The relative noise was calculated using the following formula: (Impedance in Faraday cage-Impedance in the open air)/(Impedance in Faraday cage). **(B)** Relative noise in phase shift plotted as a function of frequency. **(C)** For validation, the same test was repeated using a dummy cell with a known resistance and capacitance values. As can be seen in the relative noise plot, the same trend can be observed with the noise in the impedance, suggesting that this may be a systemic error.

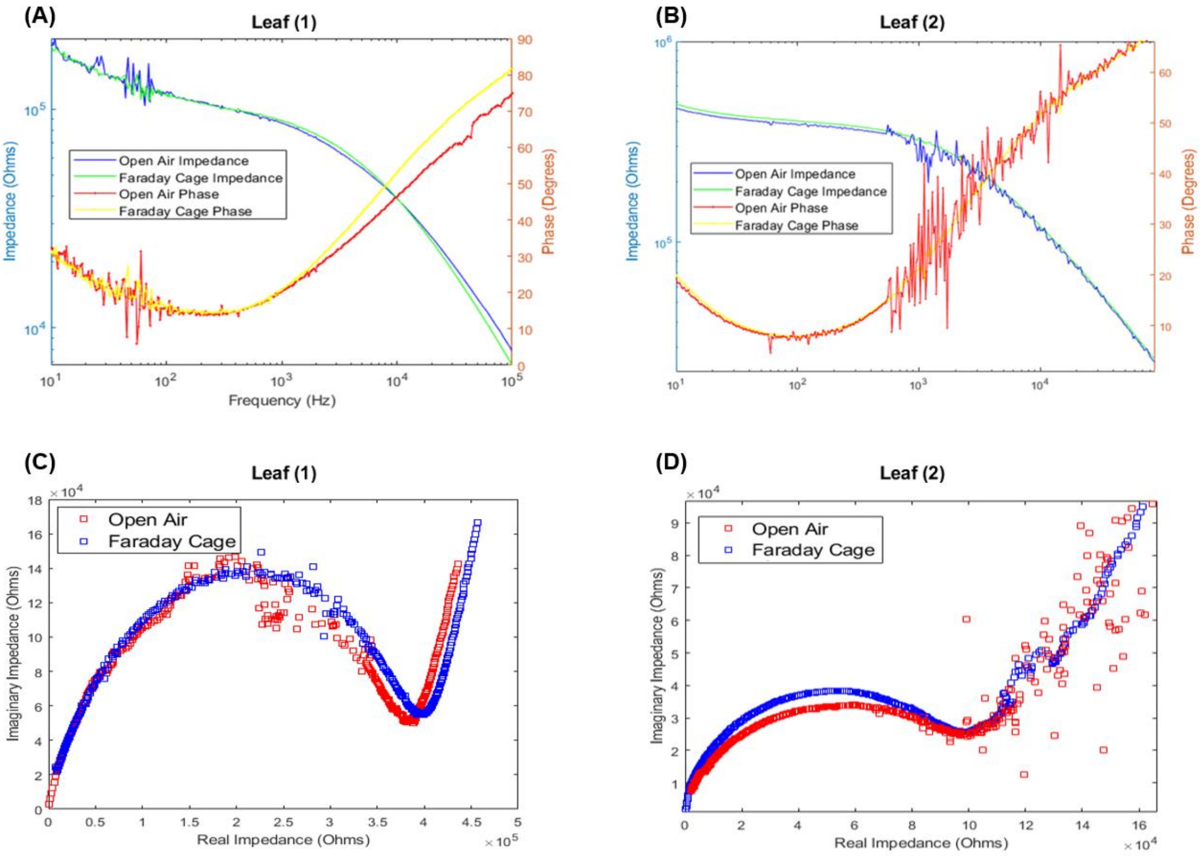

Figure S12. Bode plots for impedance recorded in Faraday cage (green line for impedance and yellow line for phase) and the open-air (blue line for impedance and red line for phase) by MNs-equipped sensor on two leaves **(A)**, **(B)** and their associated Nyquist plots **(C)**, **(D)**.

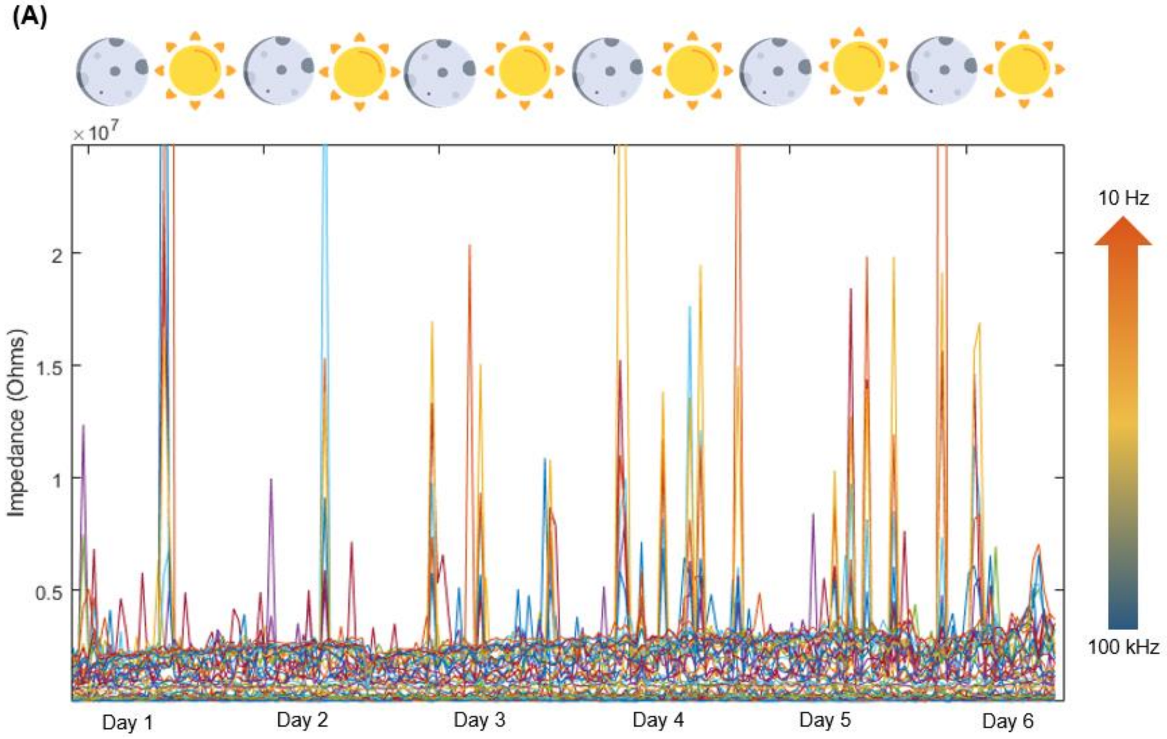

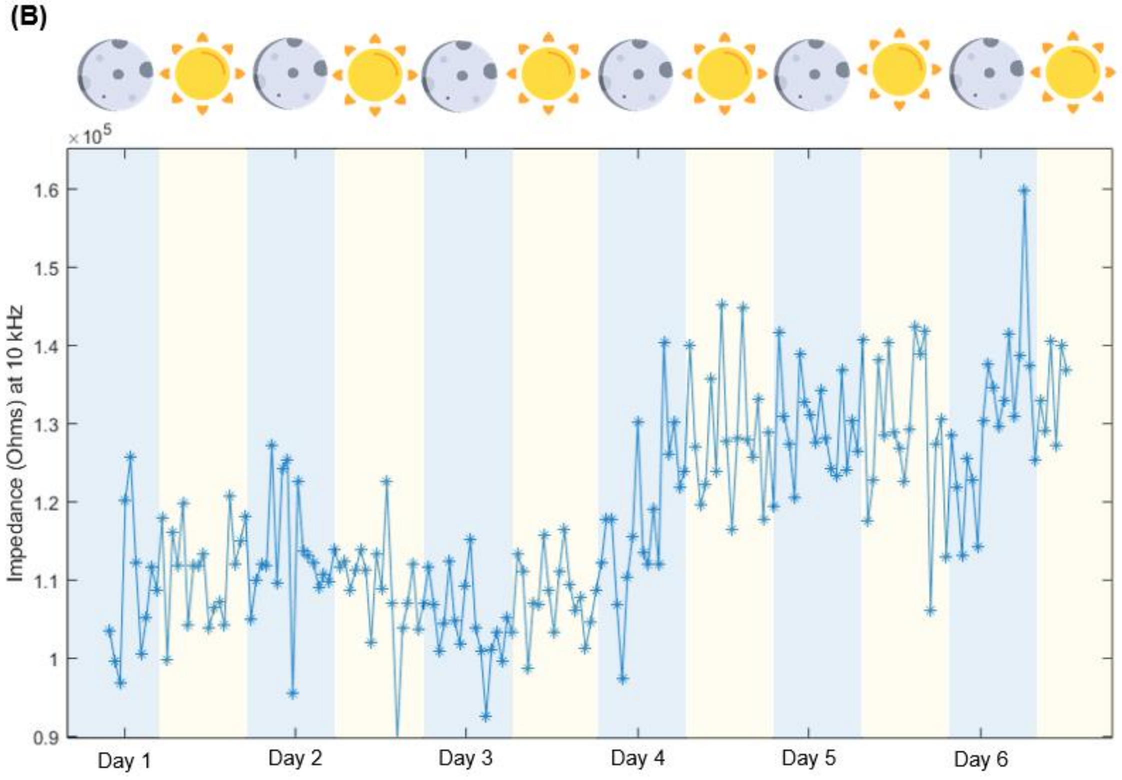

Figure S13. (A) A plot of the impedance of a plant leaf at a variety of frequencies plotted over time. The impedance spectra reported in the figure were acquired using planar electrodes. The frequencies are plotted in descending order from bottom to top as the impedance is higher at low frequencies. **(B)** The impedance values at a frequency of 10 kHz plotted over 6 days for an *Arabidopsis thaliana* specimen using planar electrodes. The shading indicates the lighting condition, where the yellow shade is for the day, and the blue shade is for the dark.

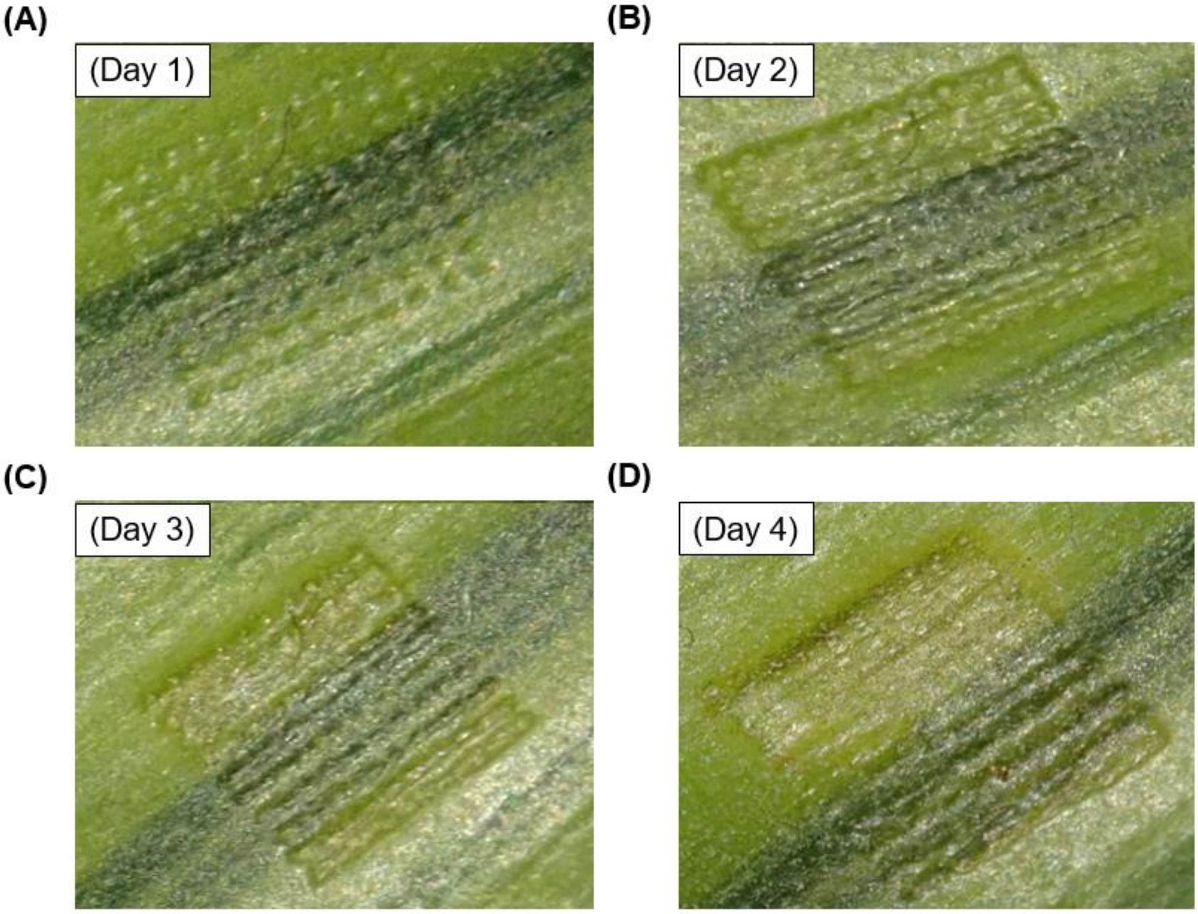

Figure S14. Magnified images (4x) showing the puncture site on an *Arabidopsis thaliana* specimen upon removal of the MNs after **(A)** one, **(B)** two, **(C)** three, and **(D)** four days.

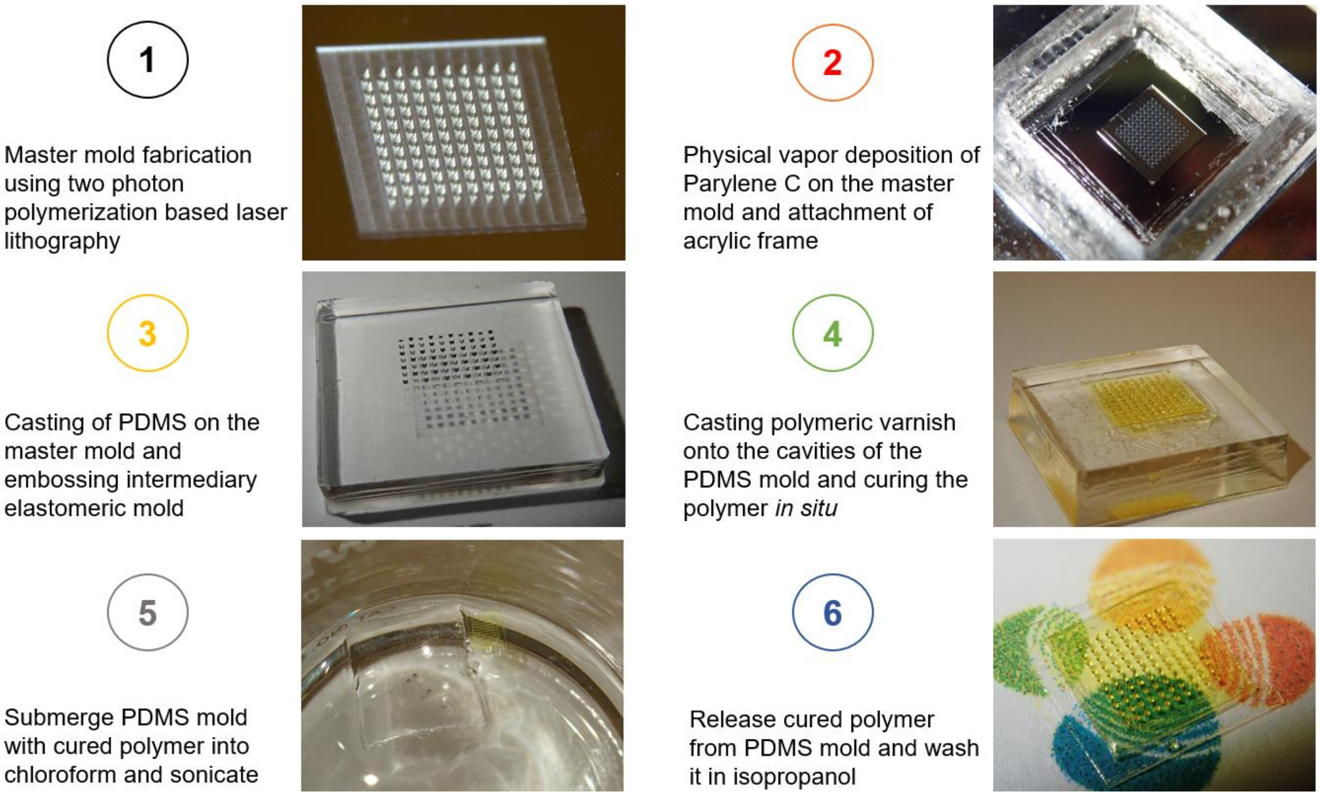

Figure S15. Documentation of micromolding and release strategy employed in the study for the fabrication of SU-8 and polyimide MNs.

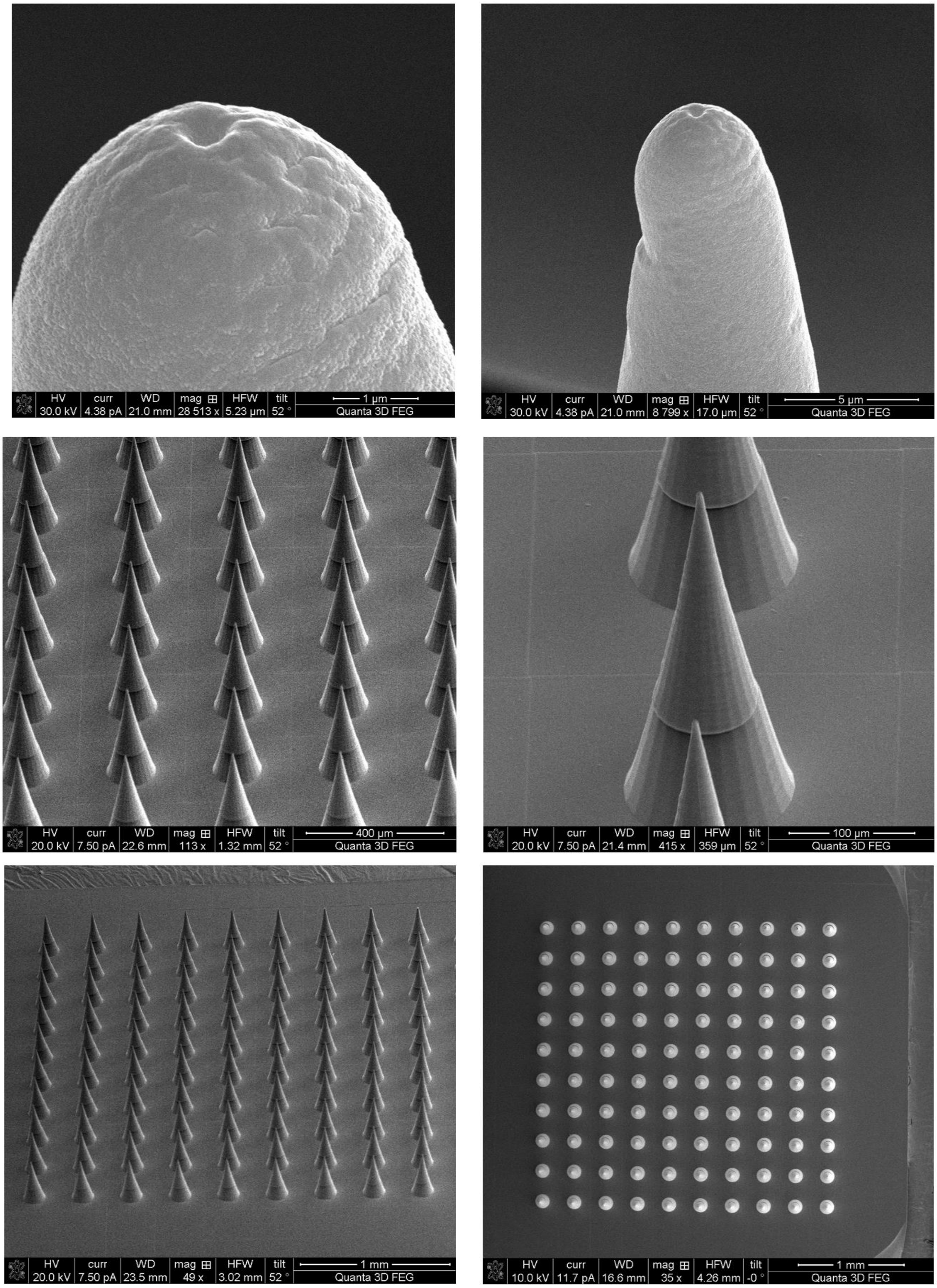

Figure S16. Additional SEM images of polyimide MNs used for EIS-based sensing.

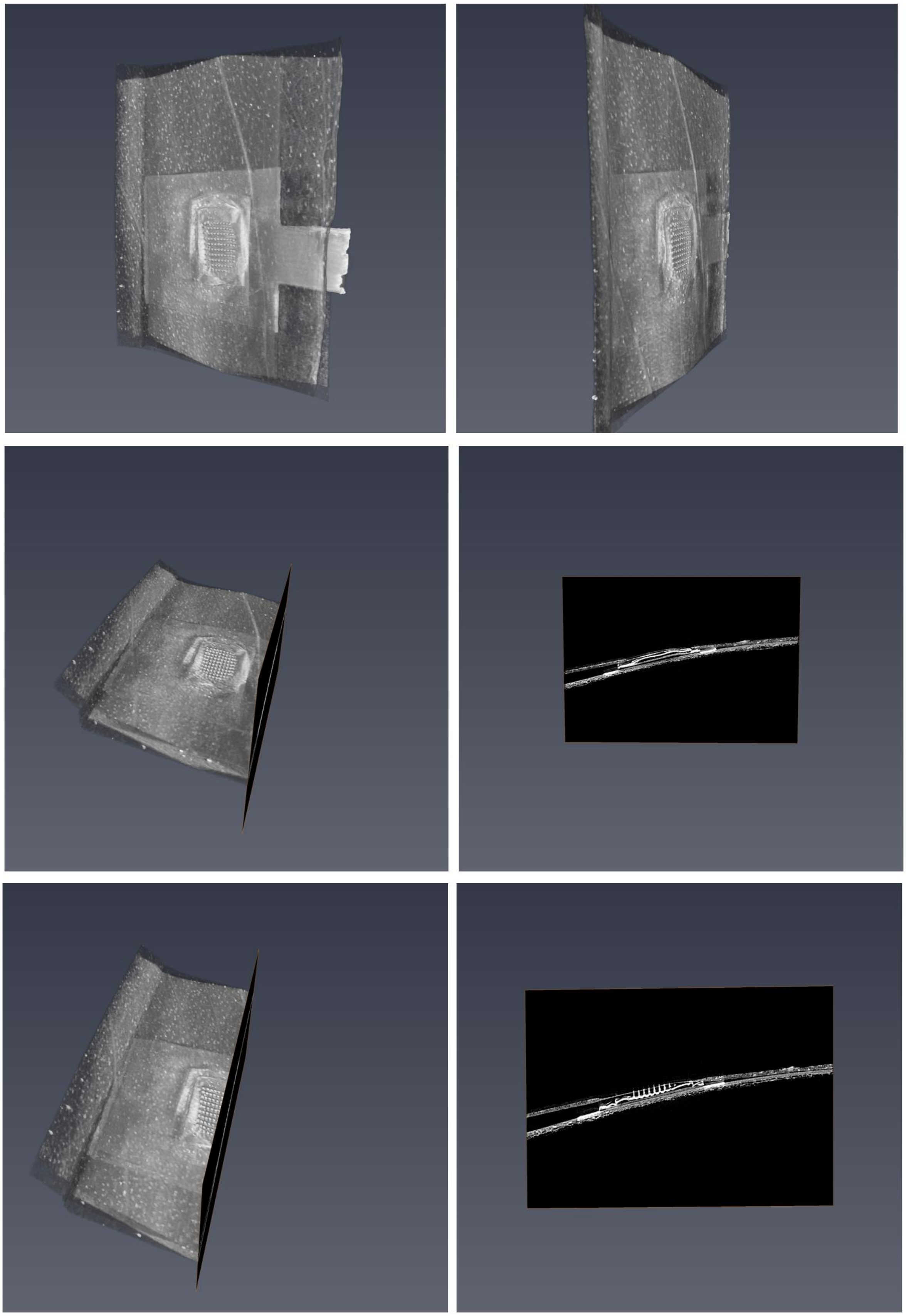

Figure S17. Additional 3D X-ray cross-sectional reconstruction images of MNs inserted into the leaf.

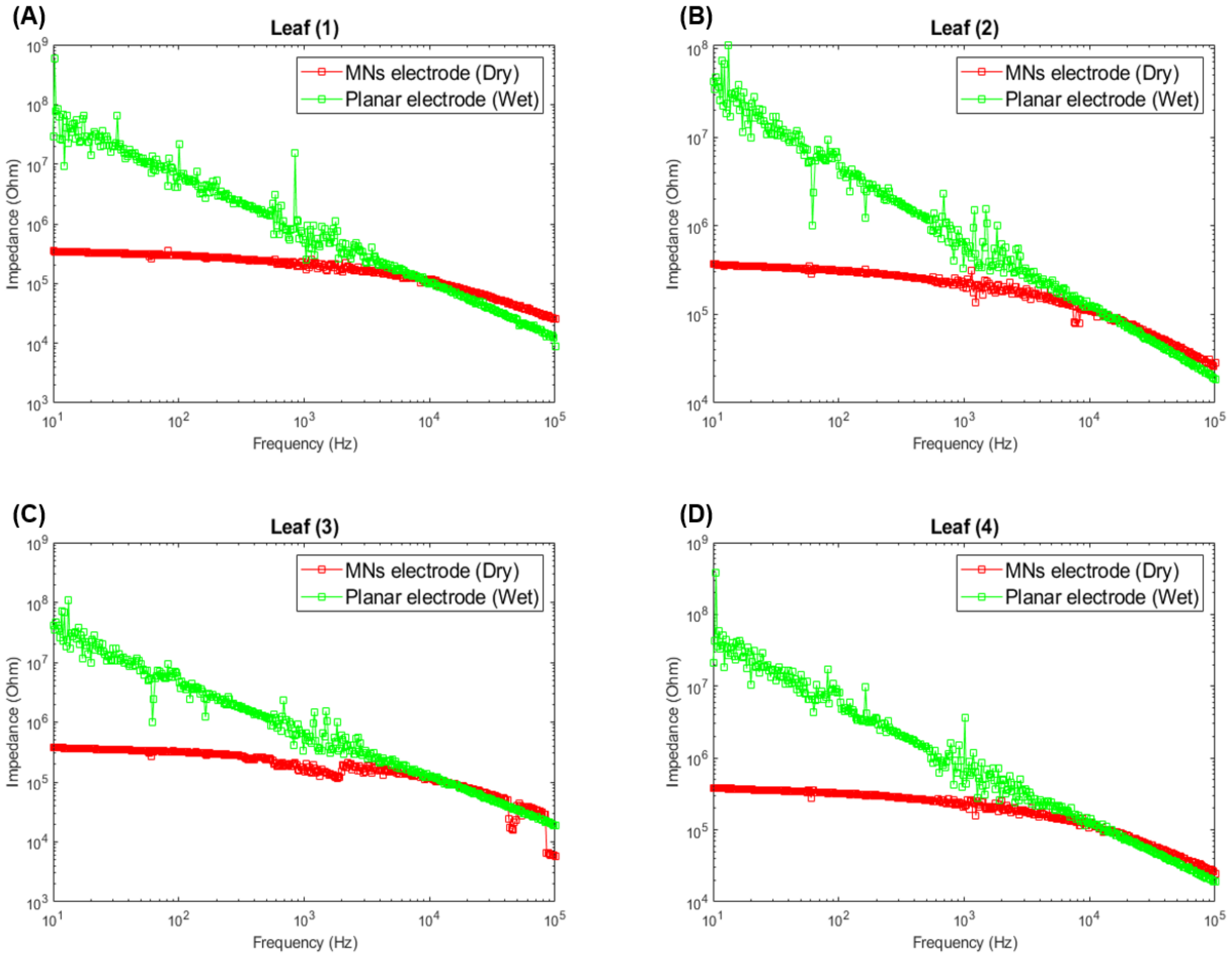

Figure S18. Impedance recorded for freshly cut leaf by the planar sensor with a wet (green) contact and by MNs-equipped sensor (red) on different leaves **(A), (B), (C),** and **(D).**

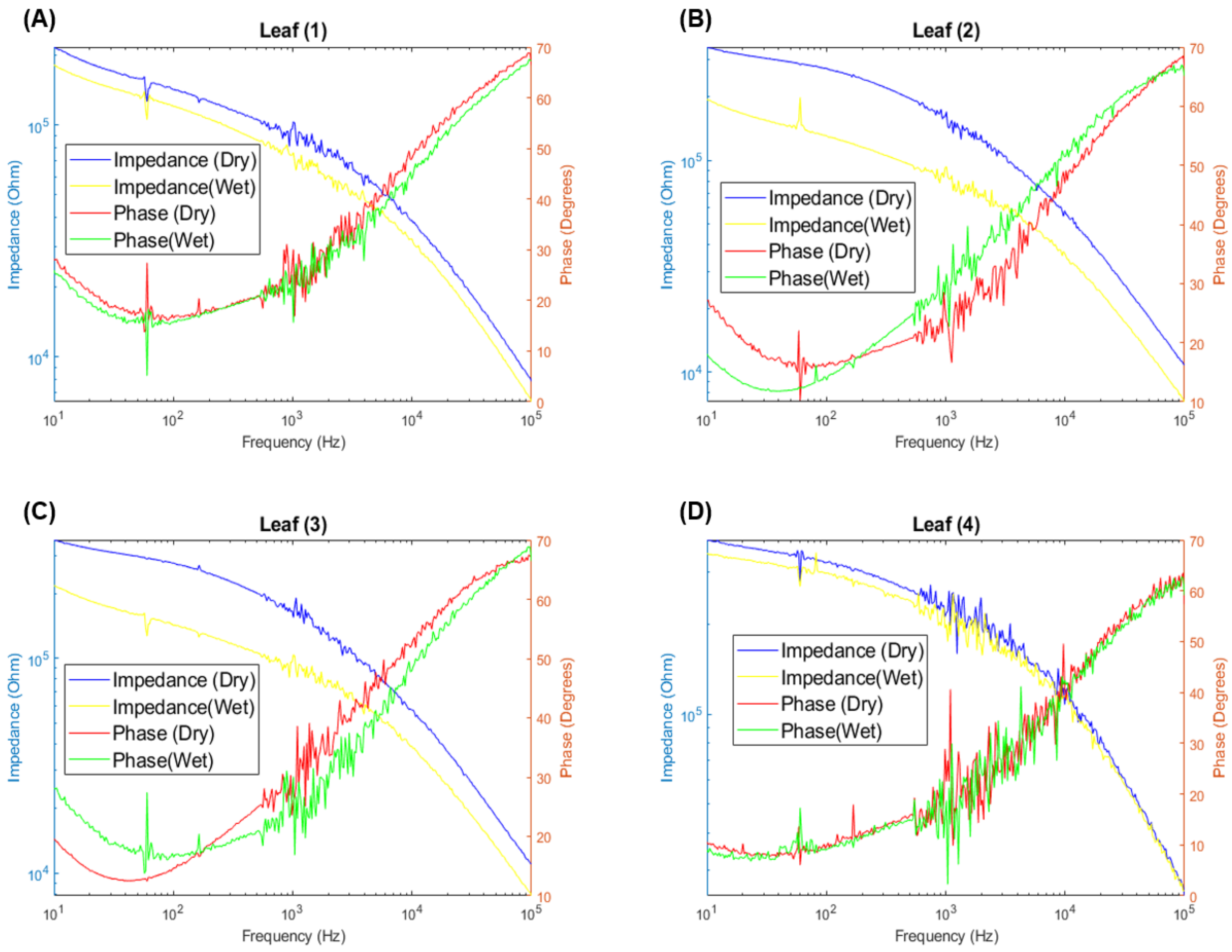

Figure S19. Bode plots for impedance recorded by MNs-equipped sensor for plant specimens **(A)**, **(B)**, **(C)**, and **(D)** while hydrated (yellow line for impedance and green line for phase) and dry (blue line for impedance and red line for phase).
